# Invasive DNA elements modify the nuclear architecture of their insertion site by *KNOT*-linked silencing in *Arabidopsis thaliana*

**DOI:** 10.1186/s13059-019-1722-3

**Published:** 2019-06-11

**Authors:** Stefan Grob, Ueli Grossniklaus

**Affiliations:** Department of Plant and Microbial Biology & Zurich-Basel Plant Science Center, University of Zurich, Zollikerstrasse 107, 8008 Zurich, Switzerland

**Keywords:** 3D nuclear organization, *Arabidopsis*, Gene silencing, Paramutation, Transgene, *KNOT*

## Abstract

**Background:**

The three-dimensional (3D) organization of chromosomes is linked to epigenetic regulation and transcriptional activity. However, only few functional features of 3D chromatin architecture have been described to date. The *KNOT* is a 3D chromatin structure in *Arabidopsis*, comprising 10 interacting genomic regions termed *KNOT ENGAGED ELEMENTs* (*KEEs*). *KEEs* are enriched in transposable elements and associated small RNAs, suggesting a function in transposon biology.

**Results:**

Here, we report the *KNOT*’*s* involvement in regulating invasive DNA elements. Transgenes can specifically interact with the *KNOT*, leading to perturbations of 3D nuclear organization, which correlates with the transgene’s expression: high *KNOT* interaction frequencies are associated with transgene silencing. *KNOT*-linked silencing (KLS) cannot readily be connected to canonical silencing mechanisms, such as RNA-directed DNA methylation and post-transcriptional gene silencing, as both cytosine methylation and small RNA abundance do not correlate with KLS. Furthermore, KLS exhibits paramutation-like behavior, as silenced transgenes can lead to the silencing of active transgenes in *trans*.

**Conclusion:**

Transgene silencing can be connected to a specific feature of *Arabidopsis* 3D nuclear organization, namely the *KNOT*. KLS likely acts either independent of or prior to canonical silencing mechanisms, such that its characterization not only contributes to our understanding of chromosome folding but also provides valuable insights into how genomes are defended against invasive DNA elements.

**Electronic supplementary material:**

The online version of this article (10.1186/s13059-019-1722-3) contains supplementary material, which is available to authorized users.

## Background

**Fig. 1:**
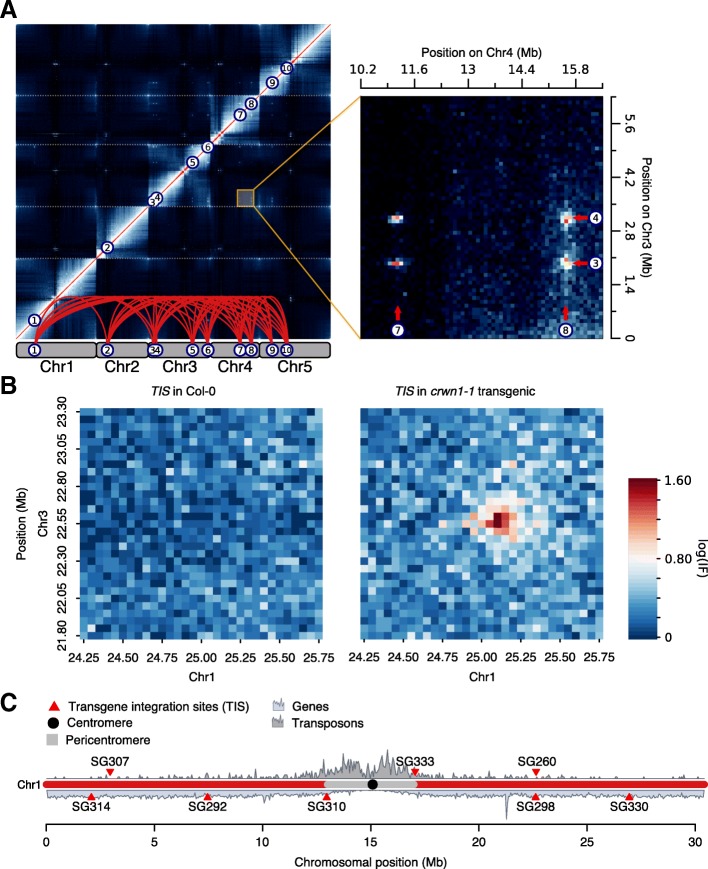
Novel *KNOT* interactions in transgenic plants. **a** Left: Hi-C interactome of *Arabidopsis thaliana*. The *KNOT* is represented by network of long-range *cis*- and *trans*-contacts found between all *Arabidopsis* chromosomes (see also Additional file 1: Table S1). **b** Hi-C interaction data representing interaction frequencies (IFs) between genomic regions on chromosome 1 (Chr1) (*TIS*_*crwn1-1*_, Chr1: 25151270–25156323) and Chr3 (*KEE6*, Chr3: 22560488–22580488). Ectopic 3D contacts can be observed between the *TIS* and *KEE6*. Left: Col-0 wild-type, Right: *crwn1-1* T-DNA insertion mutant. Interaction frequencies are pooled into 50-kb genomic bins (see also Additional file 1: Figure S1A-B). **c** Representation of *TISs* on Chr1 investigated in this study. Gene and transposon density are shown to facilitate the distinction of euchromatic and heterochromatic regions

Genome organization encompasses the linear genome, the epigenome, and its three-dimensional architecture (3D genome). In contrast to the first two organizational levels, our understanding of the functional roles of the 3D genome is rather poor. Chromosome conformation capture (3C) technologies [1] have facilitated its exploration, implicating it in transcriptional regulation [2], replication [3], and senescence [4]. We previously proposed a role of the 3D genome in transposon biology in *Arabidopsis* [5]: Ten *KNOT ENGAGED ELEMENTs* (*KEEs*) (aka IHIs [6]), transposable element (TE) insertion hotspots enriched in associated small RNAs (sRNAs), contact each other to form a nuclear structure termed the *KNOT* (Fig. 1a and Additional file 1: Table S1). The *KNOT* is conserved in plants, found in both dicots and monocots, and a potentially analogous structure may be formed by *Drosophila* piRNA clusters [5, 7].

Invasive DNA elements, such as TEs, retroviruses, and transgenes, not only are central to biotechnology but also play an important role in disease [8] and genome evolution [9]. Plants have evolved a balanced response to these elements, allowing for potential benefits, such as rapid adaptation to environmental challenges through controlled mobility [10]. In contrast, their uncontrolled proliferation and expression, which can lead to genome instability and potentially harmful ectopic gene expression, respectively, is counteracted by the silencing of invasive elements. With transgenes, silencing has been observed since the beginning of their use (reviewed in Kooter et al., [11]) and is of concern to both, gene technology and fundamental research. In plants, many transgenic approaches are based on T-DNA vectors [12]. However, despite their common origin, vectors used to generate transgenic plants exhibit significant differences with respect to transgene expression. Certain vectors, especially those containing viral *35S* regulatory sequences [13], such as *pROK2* used to generate the insertion lines of the SALK collection [14], become more frequently silenced than others. It is unlikely that the underlying mechanism is directly associated with these transgenes, as plants must have evolved strategies to counteract invasive elements well before plant transformation was developed. Hence, although the susceptibility to silencing differs among vectors, the underlying mechanisms are likely universal, irrespective of the variation with respect to silencing. The high frequency and variability of silencing among SALK lines make them an ideal system to study the control of invasive genetic elements. Suppression of such elements in plants has been associated with sRNA-mediated processes, leading to either transcript decay or DNA methylation and transcriptional silencing [13, 15]. Here, we introduce an alternative silencing phenomenon, *KNOT*-linked silencing (KLS), and show how transgenes and the 3D genome can reciprocally influence each other.

## Results

### Ectopic 3D contacts between *TRANSGENE INTEGRATION SITEs* and the *KNOT*

We reanalyzed previously published Hi-C data [5] obtained from mutant plants and observed novel high-frequency long-range interactions that were absent in the wild type (Fig. 1b). In the *crwn1-1* mutant [16], caused by a T-DNA insertion, these novel interactions occur between the *CRWN1* locus and several *KEEs*. Additionally, we observed an enrichment of interaction frequencies between the *TRANSGENE INTEGRATON SITE *(*TIS*) and constitutive heterochromatin of all five *Arabidopsis* chromosomes (Fig. 1b and Additional file 1: Figure S1A-B).

**Fig. 2:**
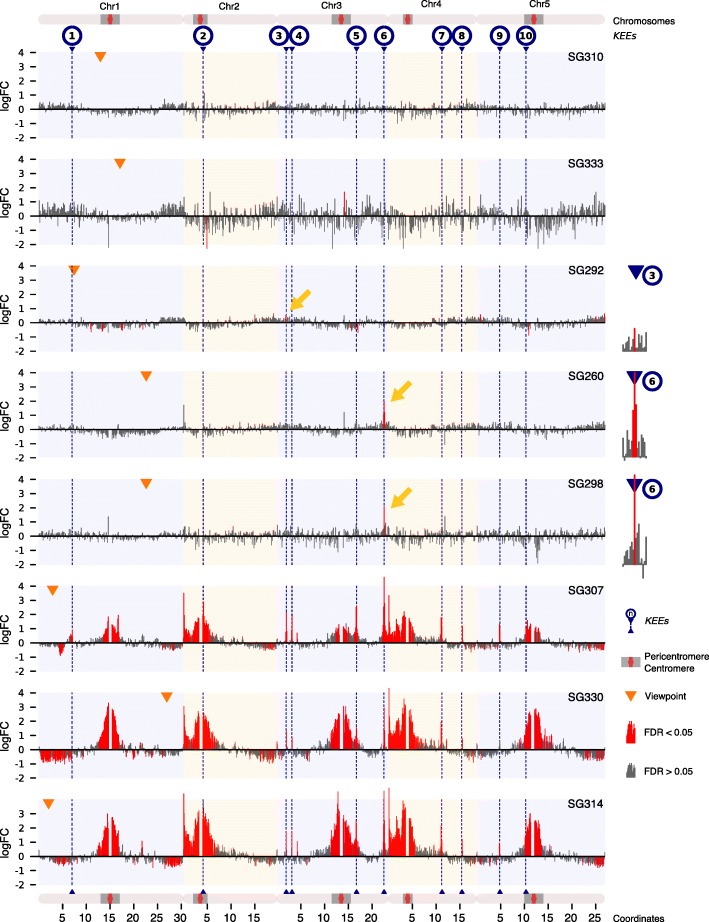
*TISs* interact with *KEEs* and pericentromeric regions. Differential analysis of 4C interactomes, including 3 wild-type and 3 transgenic 4C samples. Log2 fold changes (FC) are plotted. Gray: non-significant FC (FDR > 0.05). Red: significant FC (FDR ≤ 0.05). Orange triangles indicate viewpoints (adjacent to *TIS* on endogenous sequence). Blue triangles and dashed blue lines indicate positions of *KEEs*. Gray rectangles delineate pericentromeric regions. Interaction frequencies of single *Hind*III restriction fragments were pooled into 100-kb genomic bins. Yellow arrows indicate significant *TIS-KEE* contacts, for which magnification is given on the left

We hypothesized that transgene integration can induce ectopic *KEEs* that originate from the *TIS*, resulting in novel high-frequency contacts between the *TIS* and the *KNOT*. Thus, transgene integration may disturb the endogenous 3D organization of the *TIS*. To test this hypothesis, we performed 4C experiments in 8 independent, publicly available transgenic SALK lines, setting the viewpoint at the respective *TIS* (Fig. 1c). In parallel, we generated 4C interaction profiles of the same viewpoints in Columbia-0 (Col-0) wild-type plants and statistically evaluated differences between transgenic and wild-type 4C profiles (Fig. 2). Between transgenic and wild-type lines, differential interaction analysis revealed significant differences (FDR < 0.05), predominantly coinciding with *KEEs* (6 of 8 transgenic lines) (Fig. 2). However, the magnitude of perturbation in the 4C profile differed considerably among lines. Three of them (SG260, SG292, and SG298) exhibited a significant change in interaction frequencies only with respect to one individual *KEE* (*KEE3* for SG292 and *KEE6* for SG260 and SG298, respectively). Other transgenic lines (SG307, SG314, and SG330) showed more severe perturbations of their 4C profile. We detected ectopic high-frequency contacts with most *KEEs* and with pericentromeric regions of all chromosomes, reminiscent of the initial observation in *crwn1-1* (Fig. 2 and Additional file 1: Figure S1A-B). Thus, transgene integration does not solely result in the insertion of additional genetic material but can also perturb the 3D organization of the *TIS* in a specific manner. The absence of increased *TIS*-pericentromere interactions observed in SG260, SG292, and SG298 indicates that novel *TIS-KEE* interactions are not a consequence of *TIS* dislocation towards the pericentromere.

To assess whether the ectopic *KEE6*-*TIS* interactions coincide with decreased interaction frequencies between *KEE6* and other *KEEs*, we analyzed *KEE6-KNOT* and *KEE6-CRWN1* interaction frequencies in *crwn1-1* Hi-C data and other Hi-C data sets (wild-type and transgenic) [5, 17], which did not exhibit ectopic *KEE6-CRWN1* interactions. Indeed, *KEE6-KNOT* interaction frequencies were decreased in *crwn1-1*, suggesting that interactions of *KEE6* with the *KNOT* are diluted when it also contacts the transgene (Additional file 1: Figure S1E-F).

### Number of insertions may influence the strength of *TIS-KNOT* interactions

To further investigate variation in the extent of 3D genome perturbations between lines, we analyzed the number of *TIS* by Southern blotting and droplet digital PCR (ddPCR) (Additional file 1: Table S2 and Figure S2A). Transgenic lines exhibiting either no significant changes in interaction frequencies, or significant alterations with respect to single *KEEs* only, harbored single insertions (SG260, SG292, SG298, SG310, and SG333). All lines that exhibited more severe alterations in the 3D organization (SG307, SG314, and SG330) carried multiple insertions. PCR-based analysis using primers flanking the insertion sites indicated that multiple copies were inserted at a single locus. However, although not observed by Southern blotting, ddPCR, and short read sequencing data (4C data), we cannot completely exclude that additional T-DNA fragments are inserted elsewhere in the genome. The occurrence of large-scale rearrangements, such as translocations, can be ruled out as we can readily detect such rearrangements by 4C (Additional file 1: Figure S2B-D). As half of the single-insertion and all multiple-insertion lines showed high-frequency interactions with *KEEs*, transgene copy number may influence the strength but not the potential of *TIS*-*KNOT* interactions per se.

### *TIS-KNOT* 3D interactions coincide with transgene silencing

**Fig. 3:**
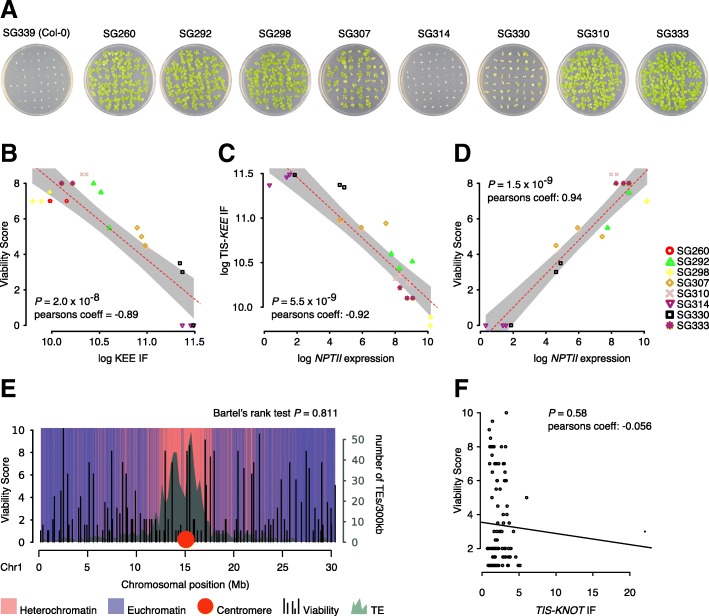
Transgene silencing by *KNOT*-linked silencing. **a** Seedlings growing on medium containing kanamycin show variable resistance (see also Additional file 1: Figure S3A). **b** Pearson’s correlation between phenotypic viability assessed by visual inspection (10—fully viable, 0—dead) in the presence of kanamycin and *TIS* interaction frequencies (IF) with *KEEs* and pericentromeres (see also Additional file 1: Table S3). **c** Pearson’s correlation between *NPTII* transgene expression and *TIS* interaction frequencies with *KEEs* and pericentromeres. **d** Pearson’s correlation between *NPTII* transgene expression and phenotypically assessed kanamycin resistance (see **b**). **e** Viability score (10—fully viable, 0—dead) of transgenic seedling populations (*n* = 30) grown on selective medium. Transgenic lines were selected by randomly choosing a homozygous SALK line (http://signal.salk.edu/cgi-bin/homozygotes.cgi) for each 300-kb genomic bin on Chr1. Numbers of transposons are indicated as a proxy for the presence of heterochromatin. Euchromatic and heterochromatic regions (purple and light red) correspond to chromatin states 1–7 and chromatin states 8–9, respectively, as previously defined [18] (see also Additional file 1: Table S4). **f** Pearson’s correlation analysis of interaction frequencies between the prospective *TIS* and the *KNOT* in the wild type and the viability score of transgenic lines with insertions at the respective *TIS*. *TIS-KNOT* interaction frequencies were calculated from Col-0 wild-type Hi-C matrices (100 kb bins) [5]

Next, we investigated whether ectopic *TIS*-*KEE* contacts affect the activity of the transgenes. The vector *pROK2*, used to generate the transgenic lines [14], harbors the *NPTII* kanamycin resistance gene. Thus, we visually assessed the viability of transgenic seedlings grown on medium containing kanamycin (Fig. 3a and Additional file 1: Figure S3A). The phenotypes were uniform in three distinct populations per transgenic genotype, stressing the robustness of the transcriptional state of the transgenes (Additional file 1: Figure S3A). Viability significantly anti-correlated with *TIS*-*KEE* interactions and was strongly reduced in lines with the highest *KNOT* interactions, phenocopying the absence of *NPTII* in the wild type (Fig. 3a, b). Lines with significantly increased interaction frequencies with the *KNOT* but not the pericentromeres, as well as lines without increased *KNOT* interaction frequencies, were not significantly affected by kanamycin, thus showing sufficient *NPTII* expression (Fig. 3a). We confirmed these results by RNA sequencing data, which revealed a significant anti-correlation between *TIS*-*KEE* interaction frequencies and *NPTII* expression (Fig. 3c) that itself significantly correlated with viability on kanamycin (Fig. 3d). As the strength of *TIS*-*KNOT* interactions negatively correlated with *NPTII* expression, we propose an involvement of the *KNOT* in transgene silencing.

Interestingly, repressive genomic neighborhoods of the *TISs* did not appear to affect either transgene expression or associated perturbations in *TIS* 3D organization: transgenes inserted into constitutive heterochromatin (SG310 and SG333) (Fig. 1c) were neither silenced nor exhibited strong 3D perturbations, whereas certain *TISs* in euchromatin showed significant perturbations and were silenced. To corroborate this observation, we grew 99 homozygous SALK lines carrying insertions distributed along chromosome 1 on selective medium, and scored their viability associated with *NPTII* expression. We did not observe decreased viability of lines that carry transgenes in repressive heterochromatin (Fig. 3e). Moreover, statistical analysis rejected a non-random distribution of viability scores, a finding supported by a previous study [19]. We cannot exclude that upon transformation, chromosomal localization may have influenced transgene expression, leading to counterselection of T-DNAs inserted into repressive environments. However, as they would not have been retrieved otherwise, all transgenes analyzed here were initially expressed in T_1_ seedlings and acquired a distinct expression state only after their initial selection. Hence, our results suggest that at least de novo silencing of transgenes is independent of the epigenetic environment of the *TIS*.

Furthermore, transgene silencing cannot be predicted based on wild-type interaction frequencies of a prospective *TIS* and the *KNOT*. Using Hi-C data from wild-type plants [5], we did not observe a significant correlation between the interaction frequencies of the prospective *TIS* with the *KNOT* and transgene silencing (Fig. 3f), indicating that the 3D organization of the prospective *TIS* does not predispose for silencing.

**Fig. 4:**
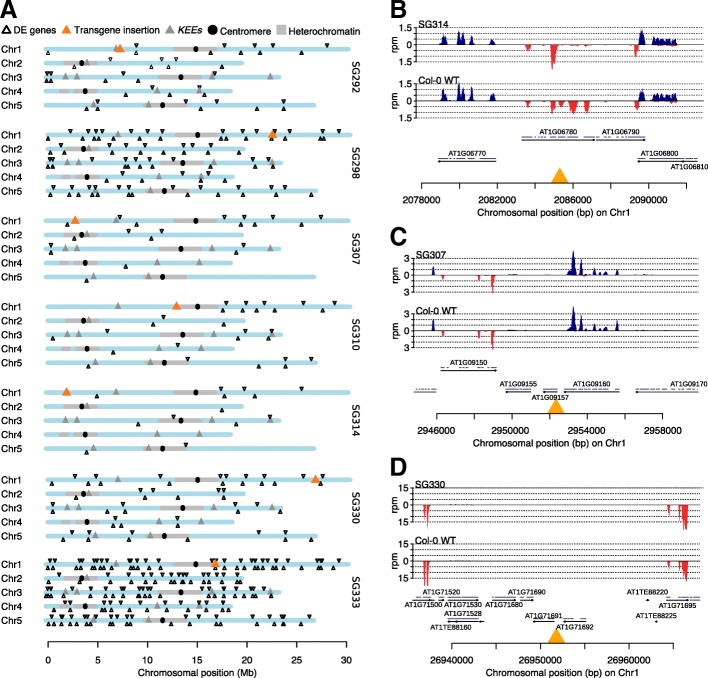
Expression profiling. **a** Differentially expressed genes between Col-0 wild-type and transgenic lines. For each line, RNA sequencing was performed in triplicate. **b**–**d** RNA sequencing coverage surrounding the transgene integration site. Top: transgenic line (pool of biological triplicates), bottom: Col-0 wild-type (pool of biological triplicates). Orange triangle depicts the transgene insertion site. Red and blue peaks represent (strand-specific) normalized read coverage (rpm, reads per million). Arrows mark genomic features. Blue lines represent exons. Read coverage was normalized to the total reads of triplicate RNA sequencing experiments. **b** Fourteen-kilobase region surrounding the *TIS* in line SG314. **c** Fourteen- kilobase region surrounding the *TIS* in line SG307. **d** Thirty-kilobase region surrounding the *TIS* in line SG330

To investigate whether perturbing the 3D organization of the *TIS* is limited to transgene expression or whether *TIS*-*KNOT* interactions also affect neighboring endogenous gene expression, we performed RNA sequencing. We analyzed triplicate mRNA from seven lines to test whether expression of genes surrounding the *TIS* differed between wild-type and transgenic lines, indicative of an effect of novel *TIS*-*KNOT* interactions. We found that transcriptional silencing is restricted to the transgene, as there was no enrichment of differentially expressed genes in the neighborhood of the *TIS* or the *KEEs* (Fig. 4a)*.* Similarly, close inspection of the transcriptional activity of neighboring genes did not reveal a significant difference between wild-type and transgenic plant lines (Fig. 4b–d).

Endogenous loci evade KLS, indicating specificity to invasive genetic elements. Furthermore, although the genomic region encompassing the *TIS* and nearby genes is folded into a repressive environment, silencing is limited to the transgene itself. Thus, a perturbation of nuclear architecture alone is not sufficient to silence gene expression and other, yet to be discovered, factors may play a role in KLS specificity.

### Transgene silencing does not require DNA methylation

Next, we aimed at putting KLS into the context of established silencing mechanisms in plants. There have been numerous previous reports on transgene silencing, and the underlying mechanisms have been deciphered [13]. Two principle mechanisms are proposed to initiate and/or maintain transgene silencing: transcriptional gene silencing (TGS) and post-transcriptional gene silencing (PTGS) lead to transcriptional arrest and mRNA degradation, respectively. Homology-dependent gene silencing, another term often used for transgene silencing, can depend on either TGS [20] or PTGS [21]. It can lead to simultaneous silencing of various homologous sequences and, hence, exhibits *trans*-silencing effects [22, 23]. SALK T-DNA lines were found to be subject to TGS, involving the accumulation of promoter-specific sRNAs and elevated levels of cytosine methylation in transgene promoters, mediated by the RNA-directed DNA methylation (RdDM) pathway [23].

**Fig. 5:**
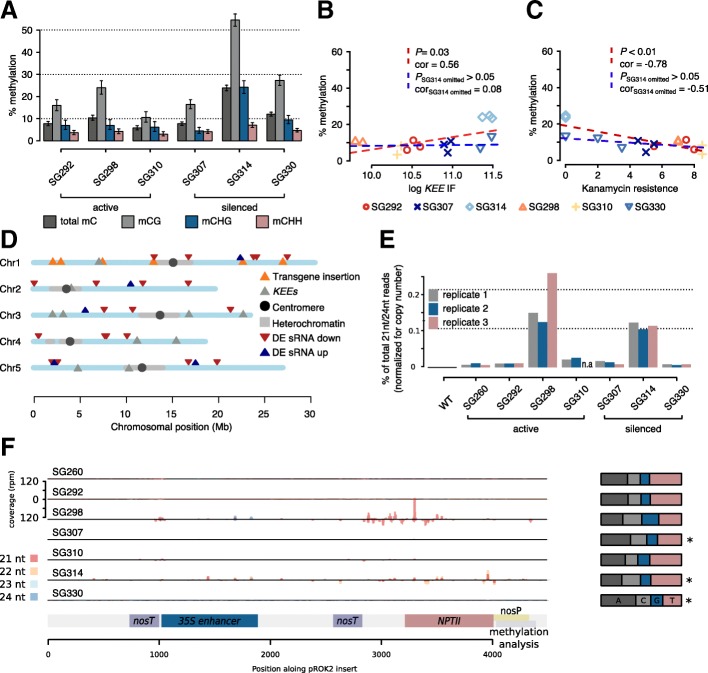
KLS is independent of canonical silencing pathways. **a** Bisulfite Sanger sequencing of *nosP* (301 bp on 3′-end of the insert). Methylation levels in all contexts significantly differed between active and silenced lines and also between individual lines (Additional file1: Table S5–6, Figure S3B-E). Error bars: Wilson 95% confidence intervals. **b** Pearson’s correlation analysis between 4C interaction frequencies with *KEEs* and pericentromeres (*KEE*-IF) and *nosP* methylation levels. Weak correlation was observed (red line). Non-significant correlation was observed when the highest methylated line (SG314) was omitted (blue line). **c** Correlation between kanamycin resistance phenotype and *nosP* methylation levels. Weak correlation was observed (red line). Non-significant correlation was observed when SG314 was omitted (blue line). **d** Differential analysis of sRNA-seq data. Genome-wide genomic bins (500 bp) exhibiting significant changes (logFC > 2; FDR < 0.01) between S- and A-lines (see also Additional file 1: Figure S4A). **e** Percentage of 21 nt and 24 nt sRNA-seq reads found within *pROK2*. For each genotype, biological triplicates were assessed (the number of reads were normalized by transgene copy number) (Additional file 1: Table S2). **f** Coverage of sRNA sequencing reads in 10 bp bins (21 nt–24 nt) mapping to the vector *pROK2.* Read numbers were normalized for *pROK2* copy number. The reads of the three biological replicates were pooled. Right: Summary of first nucleotides of the reads. Asterisks mark significant deviations from equal distribution of nucleotides (chi-square test, *P* < 0.05)

We first investigated cytosine methylation levels in the nopaline synthase promoter (*nosP*) driving *NPTII* in three active (A-lines) and three silenced lines (S-lines) by Sanger sequencing after bisulfite conversion (Fig. 5a). In average, S-lines showed elevated cytosine methylation levels and weak correlations with both, kanamycin sensitivity and *KEE* interaction frequencies (Fig. 5a–c and Additional file 1: Figure S3B-E). However, SG314, exhibiting significantly higher cytosine methylation levels than all other lines, had a major effect on the statistical analysis. By omitting SG314, no significant cytosine methylation enrichment in S-lines and no significant correlation between either transgene silencing or *KEE* interaction frequencies and methylation levels were observed (Fig. 5a–c). In all transgenic lines, including SG314, the overall *nosP* cytosine methylation levels were lower than expected for RdDM and comparable or below average genomic methylation levels [24–27]. In summary, although one transgenic line (SG314) exhibited elevated cytosine methylation levels, which may be associated with RdDM, other silenced lines (SG307 and SG330) showed low methylation levels, indistinguishable from those of active transgenes. Therefore, although not individually tested for each investigated transgene, we conclude that cytosine methylation is not generally required for the silencing of the investigated transgenes and that methylation-dependent TGS, such as RdDM, is not a prerequisite for KLS. Consistent with these results, methylation-independent transcriptional gene silencing has previously been reported [28].

### sRNA abundance does not correlate with KLS

To assess a possible involvement of sRNAs in KLS, we conducted sRNA sequencing. First, we analyzed the abundance of sRNA mapping to the *pROK2* transgene. In case of a significant involvement of sRNAs in silencing the investigated transgenes, and thus KLS, we expected to find high levels of associated sRNAs in S-lines and low levels in A-lines. We detected sRNAs associated with *pROK2* in all transgenic lines, although to variable extents (Fig. 5e, f). In accordance with our cytosine methylation analysis, sRNAs were abundant in SG314, yet no general correlation between sRNA levels and transgene silencing was found. By normalization of sRNA reads to transgene copy number, an A-line (SG298) even exhibits the highest abundance of sRNAs in all lines analyzed (Fig. 5e, f). Additionally, both the silenced lines SG330 and SG307 showed indistinguishable sRNA levels from A-lines (SG260, SG292, SG310). We conclude that sRNAs are neither sufficient nor necessary to silence these transgenes. In summary, our findings suggest that neither DNA methylation nor sRNAs play a primary role in silencing the investigated transgenes and that KLS does not depend on RdDM-related TGS.

To perform a genome-wide analysis of sRNA abundance in the investigated lines, sRNA reads were binned to 500 bp genomic regions and subsequently analyzed to detect loci of differential sRNA association (Additional file 1: Figure S4A). The sRNA profiles of active and silenced transgenic lines were very similar, identifying only few distinct differential loci (Fig. 5d). An analysis of genomic features overlapping the identified differential loci did not reveal obvious candidate factors involved in transgene silencing. We subsequently compared the identified differential sRNA loci with differentially expressed genes obtained from the mRNA-seq experiment using the same contrast (active *vs.* silenced transgenic lines), and no overlap between the two data sets was found. Similarly, analysis of the differentially expressed genes of this contrast did not provide candidates associated with transgene silencing. Our results suggest that sRNAs do not appear to be directly involved in silencing the investigated transgenes.

**Fig. 6:**
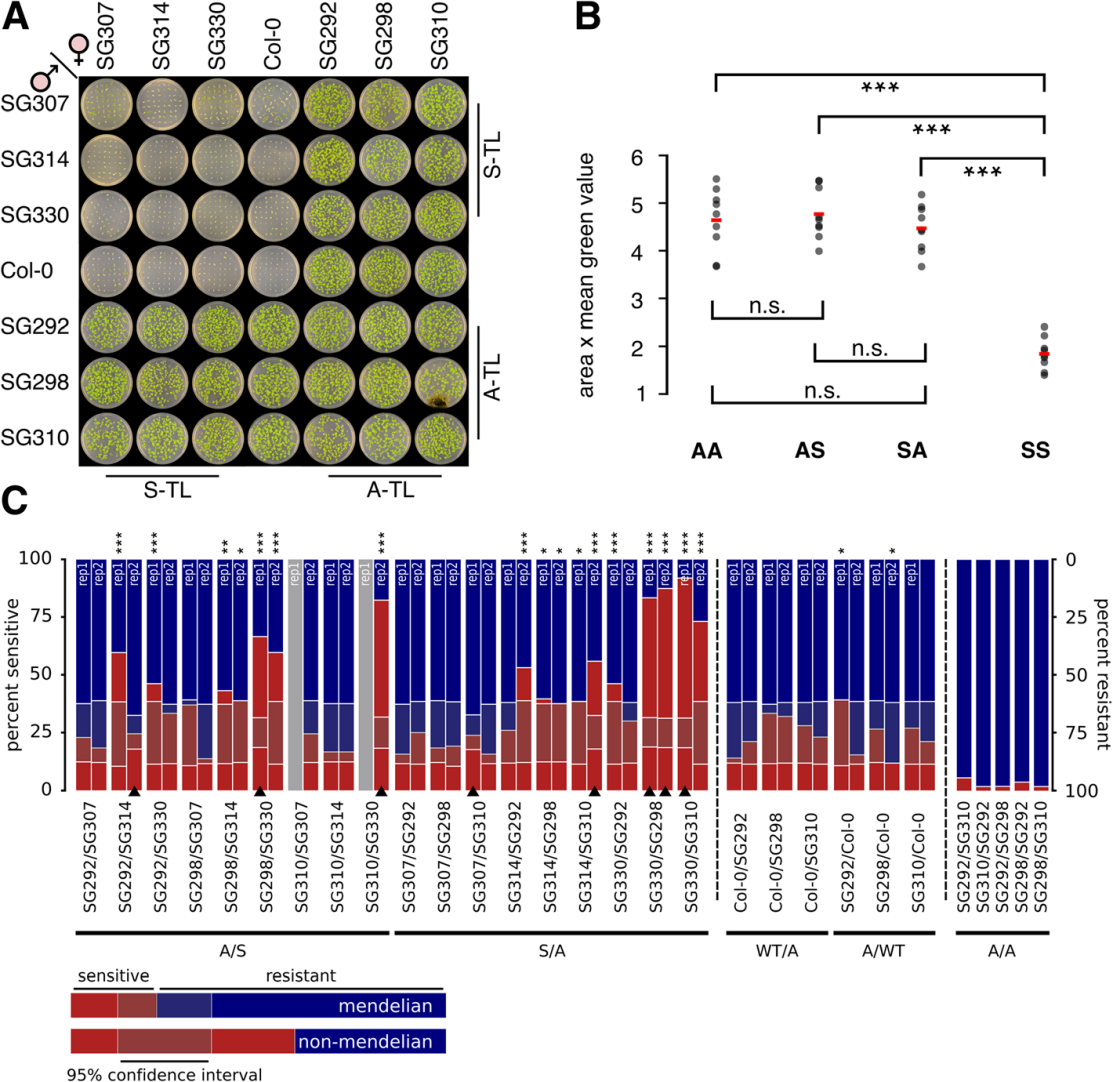
*Trans*-silencing of transgenes. **a** Reciprocal crosses between silenced (S-TL) and active (A-TL) transgenic lines. Images were acquired from 14-day-old seedlings. **b** Area and mean “green” value were assessed by ImageJ. Student’s *t* tests were performed to assess significant differences between all quarters (SS, AA, AS, SA) of the diallel cross (FDR_*SSvsAA*_ = 2.7 × 10^−7^, FDR_*SSvsSA*_ = 1 × 10^−8^, FDR_*SSvsAS*_ = 1 × 10^−8^, FDR_*AAvsSA*_ = 0.87, FDR_*AAvsAS*_ = 0.88, FDR_*SAvsAS*_ = 0.47) (Additional file 1: Table S7). **c** Segregation in F_2_ seedlings. Chi-square tests were performed to test for deviation from Mendelian segregation (null-hypothesis 0.25/0.75 (sensitive/resistant), *0.05 > *P* ≥ 0.01, **0.01 > *P* ≥ 0.001, ***P < 0.001). Confidence interval indicates the range in which Mendelian segregation cannot be rejected. Bars with black triangles stem from pooled data of 4 individual F_1_ siblings (*n* = up to 4 × 52 seedlings), non-marked bars stem from mixed seeds of the 4 siblings (*n* = up to 52 seedlings). Gray bars: data not available (Additional file 1: Table S8, Table S9, Figure S4B)

By performing an alternative experiment, we aimed to independently confirm that sRNAs are not a prerequisite of KLS. Specifically, we used a genetic approach to test whether PTGS is involved in KLS. As PTGS involves sRNAs that lead to mRNA decay, it can silence genes in *trans*. Thus, the progeny of a cross between an S- and an A-line should be at least partially silenced, as transgenes identical in sequence are present in both parental lines, such that mRNA from both transgenes should be affected by the same sRNAs. We performed reciprocal crosses using seven parental lines: one wild-type, three S-lines (SG307, SG314, SG330), and three A-lines (SG292, SG298, SG310) (Fig. 6a). This resulted in 8 progeny groups, either derived from two S-lines (SS), two A-lines (AA), two groups of progenies with parents of converse transcriptional state (SA and AS), and 4 groups of hemizygous transgenic progeny. We assessed their viability reflecting *NPTII* expression by growing F_1_ seedlings on medium containing kanamycin and by measuring the area and mean green fraction intensity of imaging data (Fig. 6a, b). The transgene expression state behaved as a heritable dominant trait (Fig. 6a). SS progeny, lacking *NPTII* expression, exhibited significantly reduced viability compared to all other groups, whereas SA, AS, and AA groups did not significantly differ from each other (Fig. 6b). Thus, in F_1_ seedlings, KLS behaves as a recessive trait with Mendelian inheritance. This excludes the involvement of diffusible sRNAs acting in *trans*, suggesting that PTGS is unlikely involved in KLS.

### KLS shows paramutation-like features

To assess whether the F_2_ generation also follows Mendelian segregation, we cultivated progeny of the above-described crosses on non-selective medium and allowed four plants of each F_1_ population to self-fertilize. We then analyzed the segregation in response to kanamycin in the F_2_ seedling populations. Assuming Mendelian segregation, double hemizygous F_1_ plants containing a silenced and active *NPTII* transgene are expected to produce 25% kanamycin-sensitive offspring (Additional file 1: Figure S4D). Employing PCR-based genotyping, we could confirm genetic Mendelian segregation for both transgenes (Additional file 1: Table S10). However, phenotypically, we observed a deviation from Mendelian segregation in a large fraction of the F_2_ populations, manifested in significantly higher proportions (up to 92%) of kanamycin-sensitive seedlings (Fig. 6c and Additional file 1: Figure S4B). The observed phenotypic segregation distortion indicates that a large fraction of parentally active transgenes underwent de novo silencing, a process reminiscent of paramutation [29]. In support of our observation, *trans*-silencing between transgenes has been observed before [22, 23]. Importantly, during the entire crossing procedure, the *trans*-silencing effect depends on the initial presence of a silenced transgene, as F_2_ seedling populations derived from AA crosses did not exhibit *trans*-silencing phenotypes (Fig. 6c). However, genotyping and subsequent quantification of *NPTII* transcripts by ddPCR of single F_2_ plants revealed that the presence of the paramutagenic allele is not necessary for the paramutagenic effect in the F_2_ generation, as plants derived from AS crosses, which were homozygous for the A but lacking the S transgene, still exhibited full *NPTII* silencing (Additional file 1: Figure S4C). The observed proportions of silencing also exclude a potential dosage effect of diffusible sRNAs associated with PTGS that are produced by the parentally silenced transgene (Additional file 1: Figure S4D-E). Furthermore, the variability in silencing ratios between F_2_ lines (Fig. 6c) suggests that specific genotype combinations observed in the segregating F_2_ populations do not directly influence the *trans*-silencing potential in single F_2_ plants. In summary, transgenes silenced by KLS show a paramutation-like behavior, but their initial silencing is not correlated with cytosine methylation and sRNAs targeting the transgene, indicating a novel phenomenon depending on 3D genome interactions.

## Discussion

### The *KNOT* is a novel player of the genome’s defense system

Our results suggest that insertion of transgenes has more profound effects on genome structure than previously anticipated, as not only genetic material is added, but also the 3D architecture of the *TIS* can be severely perturbed. These alterations have a profound impact on the transgenes themselves, as architectural perturbations can clearly be associated with the expression state of the transgenes. Importantly, the observed perturbations are not random. Moreover, we detected specific ectopic interactions with the *KNOT*, suggesting its involvement in the nuclear defense system against invasive genetic elements.

### KLS does not depend on canonical silencing pathways

In our studies on the nature of KLS, we could not find strong evidence for an involvement of either PTGS or canonical TGS, suggesting that KLS is at least initially independent of these silencing mechanisms. In support, a previous study showed that the number of *KEEs* is not reduced in mutants leading to the de-repression of silenced genes [6]. The epigenetic marks affected in these mutants include repressive histone modifications, such as H3K27me3 (*clf;swn* double mutant) and H3K9me2 (*suvh4;suvh5;suvh6* triple mutant); DNA methylation (*ddm1*, *met1*, and *cmt3* mutants); and epigenetic processes affecting silencing by other means (*mom1* mutant). This suggests that epigenetic marks commonly associated with gene silencing, such as H3K9me2, H3K27me3, and cytosine methylation, are not necessary for interactions among *KEEs*. Hence, an involvement of these canonical repressive marks in the recruitment of T-DNAs to the *KNOT*, thereby initiating KLS, is unlikely. Interestingly, in many of these mutants, an identical set of ectopic *KEEs* can be observed in apparently pre-defined positions, which show a significant enrichment of *VANDAL6* and *ATLANTYS3* TEs, both of which are highly enriched in the ten canonical *KEEs* (Additional file 1: Figure S4F). This finding suggests that inactive *KEE* regions exist in the genome, whose functional activation may rely on active transcription of TEs.

### KLS is a dynamic process

We observed that *TIS-KNOT* interactions alone are insufficient for transgene silencing, which only occurs in lines that also acquired high-frequency *TIS*-pericentromere interactions. We hypothesize that *TIS-KNOT* interactions may initiate transgene silencing, which could then be followed by a secondary alteration of the *TIS*’ 3D organization, leading to a tight association with constitutive heterochromatin and complete silencing of the transgene. Although not yet observed at the time of writing, continuous growing of lines showing exclusively *TIS-KNOT* interactions, such as SG298 and SG260, over several generations may corroborate this hypothesis.

*KEEs* vary among each other with respect to their chromosomal position and epigenetic characteristics [5]. Whereas a subset of *KEEs* originate from heterochromatic regions and exhibit contact frequency biases towards other heterochromatic regions, the majority of *KEEs* are situated in euchromatic chromosome arms, accompanied by more frequent contacts with euchromatin (Additional file 1: Figure S4G). *KEE6*, which plays the most prominent role in ectopic *TIS-KEE* contacts (Fig. 2), equally interacts with hetero- and euchromatic regions (Additional file 1: Figure S4G), despite its localization on a euchromatic chromosome arm. We speculate that *KEE6* may shuttle between both hetero- and euchromatin and, hence, may represent a key *KEE* in KLS.

KLS shows a paramutation-like behavior, whereby the transcriptional state of one transgene can be transferred to another. The KLS *trans*-silencing activity differs from classical paramutation, as it affects non-homologous loci and seems to depend on passage through an additional generation, the latter having also been observed for other transgenes with paramutation-like behavior in *Arabidopsis* [30]. Furthermore, the maintenance of the repressed paramutated state in maize requires factors involved in the biogenesis of 24 nt long sRNAs, which are homologous to components of the RdDM pathway in *Arabidopsis* [31]. Similarly, sRNAs have previously been implicated in homology-dependent *trans*-silencing in *Arabidopsis* [22, 23]. In contrast to these findings, sRNAs do not seem to play a determining role in KLS, making their involvement in KLS-related *trans*-silencing unlikely. Importantly, 3D chromosome folding has previously been reported to be involved in paramutation, specifically in *cis*-interactions between the maize *b1* locus affected by paramutation and its regulatory sequences [32]. Similarly, 3D architecture has been found to play a role in paramutation-like interactions between *Polycomb* group response element-containing (trans)genes in *Drosophila* [33].

Our work may stimulate future studies elucidating the regulatory mechanisms of paramutation-like phenomena in plants, especially concerning the developmental timing of its establishment and maintenance. The observation that not all F_2_ offspring derived from a single F_1_ plant exhibit de novo silencing of the transgene suggests that KLS may be established after floral induction. In contrast, decisions on its maintenance may occur in the vegetative shoot meristem because all F_3_ offspring from a single F_2_ plant show a uniform phenotype. However, silencing is not inherited in all F_3_ populations derived from F_2_ plants with two silenced A alleles as some pedigrees showed a reversion to the active state (Additional file 1: Figure S4H). It is worth noting that reversion was only observed in the pedigree of SG310xSG330 crosses but not SG298xSG330 crosses. In contrast to SG310, which showed no significant alteration of the *TIS*’s 3D profile, SG298 exhibited increased *TIS-KNOT* interactions (Fig. 2), which might facilitate the stable silencing of the transgene.

Transgene silencing represents an acquired epigenetic state, which is stably inherited over subsequent generations. All the transgenic lines analyzed here initially exhibited active *NPTII* transcription [14]; hence, KLS is a dynamic process, potentially established and augmented over consecutive generations. Previous reports on transgenerational epigenetic inheritance implicated DNA methylation in this process [34, 35]. Our results suggest an independent role for 3D genome organization in the transgenerational epigenetic inheritance of silenced transgenes. Although we observed tight *TIS-KEE* interactions being stably inherited over subsequent generations, we also show that KLS may contribute to the plasticity of transgenerational epigenetic inheritance through a paramutation-like *trans*-silencing mechanism and its reversion.

### Molecular mechanism underlying KLS remains to be deciphered

Very likely, KLS involves a set of protein cofactors that mediate 3D *TIS-KEE* interactions. The identification of these cofactors will be essential for a better understanding of KLS and its embedding within other nuclear processes. However, this search will be challenging due to the technical inaccessibility of KLS phenotypes, such as *TIS-KEE* interactions, for large-scale genetic screening.

KLS represents a previously uncharacterized phenomenon to defend the genome against invasive DNA elements. Hence, KLS not only is important for a basic understanding of gene regulation in the context of the 3D genome but also is of great interest to plant biotechnology, as transgene integration may have a larger impact on genome architecture than previously thought.

## Conclusions

Mobile invasive DNA elements can threaten proper genome function. Hence, their transcription is regulated and can be shut down by cellular processes known as gene silencing mechanisms. We here present a novel aspect of gene silencing, which is linked to the *KNOT*, a specific 3D chromosomal structure. Our results suggest a functional role of 3D genome folding in the defense against invasive DNA elements. KLS appears to be independent of previously published silencing mechanism, whose hallmarks are increased DNA methylation and RNA interference. In fact, KLS may even underlie these silencing mechanisms and represent an initial stage in recognizing foreign DNA elements. Interestingly, the *KNOT* is conserved within the plant kingdom, such that KLS may represent a basal silencing mechanism common to most plants.

## Methods

### Plant material

Seeds of transgenic *Arabidodpsis thaliana* lines were acquired through the European Arabidopsis Stock Center (NASC) (http://arabidopsis.info/) (Additional file 1: Table S16). All parental lines were genotyped, and homozygous individuals were selected and selfed to produce F_1_ seeds. Plants were grown under long-day conditions (16 h light, 8 h dark, 22 °C day, 18 °C night). Seeds were sterilized using hypochloric acid and stratified for 2 days at 4 °C. All plant material used in this study stems from 14-day-old seedlings cultivated as previously described [36]. All analyzed *Arabidopsis* lines are in the Columbia-0 (Col-0) accession.

### 4C experiments

Generation of 4C templates was performed as previously described [36]. To minimize technical biases, we placed the 4C viewpoints adjacent to the *TIS* on endogenous DNA sequence; thus, the 4C template enrichment could be performed using identical primer pairs in transgenic and wild-type 4C samples. All 4C experiments were performed in biological triplicates. Primer sequences and restriction enzymes used in the 4C experiments are indicated in Additional file 1: Table S12. 4C sequencing reads were aligned using bowtie [37] with the parameters -a -v 0 -m 25 (no mismatches allowed, multiple alignments allowed). Alignment scores, plant lineage, and 4C replicate information are summarized in Additional file 1: Table S17. Reads with multiple alignments were weighted using Rcount-multireads [38]. Weighted reads were mapped to individual restriction fragments using HiCdat [39], yielding tables describing the number of reads, and thus interaction frequencies (IFs) per individual *HindIII* restriction fragment. The IFs were subsequently allocated to non-overlapping 100-kb genomic bins. IFs were further processed using edgeR [40] to determine count data (log count per million) and to perform differential analysis between transgenic and wild-type 4C interaction profiles, comprising of 3 biological replicates each. For this, count data was normalized for library sizes (edgeR: calcNormFactors()), followed by estimating common and tag-wise dispersion (edgeR: estimateCommonDisp() and estimateTagwiseDisp()). Differential analysis was then performed using the exact test (edgeR: exactTest()). Significant differences in IFs between transgenic and wild-type data sets were defined by a false discovery rate (FDR) < 0.05 (using Benjamini-Hochberg-adjusted *P* values).

### Hi-C data and virtual 4C analysis

Previously published Hi-C interaction data [5] were processed as previously described [5]. Hi-C snapshots were taken from regions of interest using a 50-kb (Fig. 1b) and 100-kb (Fig. 1a) binning size. Virtual 3C and 4C analysis (Additional file 1: Figure S1A and Fig. 3f) was performed by extracting the genomic 100-kb bin relevant to the viewpoint of interest (*crwn1-1*: Chr1 25.1–25.2 Mb (Additional file 1: Figure S1A) or summing up Hi-C interaction frequencies between *TIS* and bins (100 kb) encompassing *KEEs* and pericentromeres (Fig. 3f; *KEE1* and the pericentromere of chromosome 1 were omitted). To determine *KEE6-KNOT* contact frequencies, IFs were extracted from previously published Hi-C matrices at 50-kb resolution [5, 17], without using distance normalization. *KEE6-KNOT* IFs were defined as contact frequencies between *KEE6* and *KEE1*, *KEE2*, *KEE3*, *KEE4*, *KEE5*, *KEE7*, *KEE8*, *KEE9*, and *KEE10*. Contact frequencies between *KEEs* and hetero- and euchromatic regions (defined in [5]), respectively, were extracted from Hi-C data of 50-kb bin size and are shown in Additional file 1: Figure S4G. Contact frequencies stemming from *cis*-contacts and contacts between *KEEs* were excluded from this analysis.

### Copy number analysis

The copy numbers of inserted transgenes were assessed using Southern blot analysis and droplet digital PCR. DNA for both types of analysis was extracted from 14-day-old *Arabidopsis* seedlings using a MasterPure DNA purification kit (Epicentre, Madison, WIS, USA).

#### Southern blot

For Southern blot analysis, genomic DNA was digested using the *HindIII* restriction enzyme (New England Biolabs, Ipswich, MA, USA). The digestion efficiency was analyzed on 1.5% agarose gel. Subsequently, the gel was washed for 10 min in 0.25 M HCl, followed by 15-min incubation in denaturation solution (1.5 M NaCl, 0.5 N NaOH) and 15-min incubation in neutralization solution (1.5 M NaCl, 1 M TrisHCl, pH 7.5). The fragmented and denatured DNA was then transferred to a positively charged nylon membrane (Roche, Basel, Switzerland) overnight at room temperature (RT). After rinsing the nylon membrane in 2× SSC buffer, the DNA was UV crosslinked (GS cross linker BioRad (BioRad, Hercules, CA, USA)). The membrane was then placed in a glass cylinder and incubated in 15 ml of hybridization solution (DIG Easy Hyb Granules, Roche, Basel, Switzerland) for 5 h at 42 °C under constant rotation. Following pre-hybridization, the membrane was incubated in 15 ml of fresh hybridization solution containing 8 μl of Salmonsperm-DNA and 5 μl of digoxigenin (DIG)-labeled probe (generated by incorporation of DIG-labeled dUTP (Roche, Basel, Switzerland)) at 42 °C overnight. The probe was generated using the following primer pair: forward primer: GTCAAGAAGGCGATAGAAGGCG, reverse primer: GCTTGGGTGGAGAGGCTATT and covers large parts of the *NPTII* gene. The next day, the membrane remaining in the glass cylinder was washed two times with W1 (2× SSC, 0.1% SDS) for 5 min at 68 °C, followed by 15-min washing in W2 (0.2× SSC, 0.1% SDS) at 68 °C, and 15 min in W3 (0.1× SSC, 0.1% SDS). Subsequently, the membrane was transferred to a plastic tray and incubated at RT in WB (100 mM maleic acid, 150 mM NaCl, 0.3% Tween-20, pH 7.5), followed by 30 min in B2 (2 g Roche Blocking Reagent (Roche, Basel, Switzerland) in 200 ml B1 (100 mM maleic acid, 150 mM NaCl, pH 7.5)). Then, 1.5 μl anti-DIG-alkaline phosphatase conjugate (Roche, Basel, Switzerland) in 50 ml B2 was added and the membrane was incubated for 30 min. The membrane was washed three times for 40 min in WB and then incubated for 5 min in B3 (100 mM TrisHCl, 100 mM NaCl, 50 mM MgCl_2_, pH 9.5). Finally, the membrane was overlaid with 6 ml of substrate solution (60 μl CDP Star (Roche, Basel, Switzerland) in 6 ml B3) and subsequently exposed in a trans-illuminator (Biorad Chemidoc XRS, (BioRad, Hercules, CA, USA)). Image acquisition was conducted after 10,000 s of exposure.

#### Droplet digital PCR

Droplet digital PCR (ddPCR) was performed to quantify *NPTII* (and thus transgene) copy number using a Biorad QX200 Droplet Digital PCR system (BioRad, Hercules, CA, USA). The concentration of *NPTII* (transgene), *FIE* (AT3G20740; endogenous single copy gene), and *LYS* (AT5G62150; endogenous single copy gene) was assessed using 2 ng of input genomic DNA. The rounded average between *NPTII*/*FIE* and *NPTII*/*LYS* ratios was finally used to determine the *NPTII* copy number. Droplet generation was performed according to the manufacturer’s protocol. Following droplet generation, the templates were amplified in a T100 thermal cycler (BioRad, Hercules, CA, USA). Fluorescence reads of the individual droplets were analyzed using Quanta Soft v1.7 (BioRad, Hercules, CA, USA). For each sample and probe, experiments were performed in technical duplicates. Primer and probe sequence information is shown in Additional file 1: Table S14. Probes were custom designed and acquired from Life Technologies (Life Technologies, ThermoFisher Scientific, Waltham, MA, USA).

### mRNA sequencing

RNA was extracted from 14-day-old *Arabidopsis* seedlings using RNeasy Plant Mini Kit (Qiagen, Venlo, The Netherlands). RNA was extracted from three F_1_ seedling populations per following parental plant lines: SG339 (wild type), SG292, SG298, SG307, SG310, SG314, SG330, and SG333. After library preparation using the Illumina Stranded mRNA RNA-seq protocol, total RNA was subjected to Illumina RNA sequencing (RNA-seq). RNA-seq reads were aligned using the subjunc [41] RNA sequencing read alignment program. The numbers of valid alignments are shown in Additional file 1: Table S18. Aligned RNA-seq reads were then weighted and mapped to individual transcriptional units (genes, TEs) using Rcount [38]. The preprocessed transcription data was analyzed using the edgeR [40] differential expression (DE) analysis program. DE was analyzed for two types of contrasts: (i) individual parental transgenic lines (using three F_1_ seedling populations) *vs*. Col-0 wild type, and (ii) all combined transgenic lines *vs*. wild type. To analyze DE, we chose a standard approach using general linearized models. After estimating common and trended (edgeR: estimateGLMCommonDisp() and estimateGLMTrendedDisp()) dispersion, we applied a gene-wise negative binomial generalized linear model (edgeR: glmfit(), followed by glmLRT()) to assess DE. *P* values were adjusted according to Benjamini-Hochberg, and DE genes exhibiting adjusted *P* values < 0.05 were scored as significant.

### sRNA-seq analysis

Total RNA was extracted from 14-day-old *Arabidopsis* seedlings using the mirVana miRNA isolation kit (Ambion, ThermoFisher Scientific, Waltham, MA, USA). RNA was extracted from three F_1_ seedling populations of the following parental lines: SG339 (Col-0 wild type; SG339A, SG339B, SG339C), SG260 (SG261, SG368, SG371), SG292 (SG335, SG337, SG369), SG298 (SG350, SG361, SG362), SG307 (SG342, SG355, SG356), SG310 (SG310, SG340), SG314 (SG346, SG355, SG356), and SG330 (SG352, SG353. SG359).

Total RNA was ligated to Illumina sequencing adapters, size selected, and subsequently sequenced on Illumina HighSeq 2500. The adapters of Illumina sequencing reads were trimmed using cutadapt (parameters: -m 17 –q 20; adapter sequence: TGGAATTCTCGGGTGCCAAGGAACTCCAGTCAC). Subsequently, the trimmed reads were filtered by aligning them against regions encompassing rRNA genes (10 kb surrounding them, Chr2,1..10000, Chr3,14194000..14204000) and tRNA genes, as well as chloroplast and mitochondrial sequences. The unaligned reads were size selected (17–30 bp) using an awk command and subsequently aligned to the *Arabidopsis* reference genome (TAIR10) using bowtie with the following parameters: bowtie –v 2 –best –m 10,000 (allowing two mismatches and up to 10,000 equally best alignments). The aligned sequencing reads were corrected for multiple alignment using Rcount-multireads and subsequently binned into 500-bp non-overlapping genomic bins (see also Additional file 1: Table S19). Differential analysis was performed using edgeR with the same parameters as described for differential RNA-seq analyses. To find genomic features associated with differential 500-bp bins, bedtools [42] intersect has been employed.

### NPTII expression by ddPCR

Total RNA was extracted using the standard Trizol RNA extraction protocol, followed by RNA purification using a Direct-zol Micro Prep kit (Zymo Research, CA, USA). To remove residual DNA, RNA samples were treated with 2 U of TURBO DNase following the manufacturer’s protocol (Invitrogen, ThermoFisher Scientific, Waltham, MA, USA). Ten microliters (ca. 1 μg) of purified RNA samples were incubated for 10 min at 70 °C with 1 μl oligodT, 1 μl RNase OUT (ThermoFisher Scientific, Waltham, MA, USA). Reverse transcription was performed using SuperScriptII reverse transcriptase, following the manufacturer’s protocol (ThermoFisher Scientific, Waltham, MA, USA). ddPCR was performed amplifying both *NPTII* transcripts and *UBC9* transcripts as internal control to normalize for different amount of input material.

### Methylation analysis

DNA from 14-day-old *Arabidopsis* seedlings was extracted using a MasterPure DNA extraction kit (Epicentre, Madison, WIS, USA). The DNA was bisulfite converted using an EpiTect Bisulfite Kit (Qiagen, Venlo, Netherlands) according to the manufacturer’s protocol. Bisulfite-converted DNA was amplified using a Kapa Library Amplification Kit (Kapa Biosystems, Wilmington, MA, USA). Primer sequences are indicated in Additional file 1: Table S15. PCR products (see also Fig. 5f) were subsequently purified from an agarose gel, cloned into the pJet1.2 cloning vector (CloneJET PCR cloning Kit, ThermoFisher Scientific, Waltham, MA, USA), and transformed into DH5α *E. coli* cells. Subsequently, the extracted vectors were subjected to Sanger sequencing. The resulting sequences were trimmed and preprocessed using BISMA (http://services.ibc.uni-stuttgart.de/BDPC/BISMA/) [43]. CG, CHG, and CHH methylation levels were assessed using Kismeth (http://katahdin.mssm.edu/kismeth/revpage.pl) [44]. Statistical analysis of the methylation data was performed as previously described [45, 46]. Methylation data of all F_1_ samples belonging to the same parental line were pooled, and subsequently Wilson’s 95% confidence interval was calculated using the R package “binom.” Chloroplast DNA does not exhibit cytosine methylation; thus, a region of chloroplast DNA was amplified, cloned, and sequenced to assess the bisulfite conversion efficiency (see also Additional file 1: Table S5 and S6).

### Kanamycin sensitivity phenotype analysis

#### Visual assessment

Kanamycin sensitivity in parental lines was analyzed by visual inspection. An experimenter unaware of the experimental design was asked to judge general viability of the seedlings using previously acquired images and rate the viability between 0 (dead) and 10 (perfectly viable) (double-blind assay).

#### Image data analysis

Images were acquired from 14-day-old seedlings grown on kanamycin-containing medium. The images were further processed and analyzed using the ImageJ image analysis software. The color images were split in red, green, and blue channels. All subsequent steps were conducted in the green channel images. To extract the area covered by seedling tissue, a gray threshold was set, which has been previously empirically defined and showed the best separation between seedling tissue and background (lower threshold 160, upper threshold 255). The total area and the mean gray value within the threshold were measured. The product between total area and mean gray value (area × mean; Additional file 1: Table S7) was used to perform statistical analysis. Assuming normal distribution of the data, we performed cross-wise *t* tests between progeny classes (progeny classes derived from crosses with the wild type were omitted). The *P* values were adjusted for multiple testing using the Benjamini-Hochberg (aka FDR) algorithm. Statistical testing was conducted using R.

#### Statistical analysis

Correlation between viability on kanamycin (as well as *NPTII* expression) and *TIS-KNOT* IFs was performed using the R-base cor.test() function. The slope and intercept were retrieved by employing a linear model using the R-base lm() function. To assess whether the chromosomal position may affect transgene expression, the viability score of 99 transgenic lines inserted into chromosome 1 were assessed visually. Subsequently, chromosome ordered viability scores were tested for randomness using a two-sided Bartels rank test.

## Additional file

Additional file 1: Supplementary tables (Tables S1–S19) and figures (Figures S1–S4). (PDF 5719 kb)

## Data Availability

4C and RNA, and sRNA sequencing data are publicly available at the Sequence Read Archive (SRA; https://www.ncbi.nlm.nih.gov/sra/) under accession SRP126992 [47]. Codes for data processing and analysis are available on Zenodo [48].

## Abbreviations

3D: Three-dimensional
4C: Circular chromosome conformation capture
A-line: Active transgenic line
ddPCR: Droplet digital PCR
FC: Fold change
IF: Interaction frequency
*KEEs*: *KNOT ENGAGED ELEMENTs*
KLS: *KNOT*-linked silencing
*nosP*: Nopaline synthase promotor
PTGS: Post-transcriptional gene silencing
RdDM: RNA-dependent DNA methylation
S-line: Silenced transgenic line
sRNA: Small RNA
sRNA-seq: sRNA sequencing
TE: Transposable element
TGS: Transcriptional gene silencing
*TIS*: *TRANSGENE INTEGRATION SITE*
