# Supplementary material for: Invasive DNA elements modify the nuclear architecture of their insertion site by KNOT-linked silencing in Arabidopsis thaliana
Source: Genome Biol. 2019 Jun 11;20:120. doi: 10.1186/s13059-019-1722-3 (PMC6560877; doi:10.1186/s13059-019-1722-3)
Supplement: Supplementary file 1 — Supplementary tables (Tables S1–S19) and figures (Figures S1–S4). (PDF 5719 kb) [file 13059_2019_1722_MOESM1_ESM.pdf]

Supplemental Figures and Tables

Invasive DNA elements modify the nuclear architecture of their insertion site by *KNOT*-Linked Silencing in *Arabidopsis thaliana*

Stefan Grob[a,1] and Ueli Grossniklaus[a,1]

[a]Department of Plant and Microbial Biology & Zurich-Basel Plant Science Center, University of Zurich, Zollikerstrasse 107, 8008 Zurich, Switzerland

Supplementary Tables

Table S1 Genomic positions of *KEEs. KEE* start and end positions are estimates based on the highest peak of interaction between *KEEs*.

KEE ID	Chromosome	Start (bp)	End (bp)	Estimated Center (bp)
KEE01	Chr1	7051324	7091324	7071324
KEE02	Chr2	4116555	4156555	4136555
KEE03	Chr3	1951581	1991581	1971581
KEE04	Chr3	3101455	3141455	3121455
KEE05	Chr3	16697396	16737396	16717396
KEE06	Chr3	22560488	22600488	22580488
KEE07	Chr4	11186537	11226537	11206537
KEE08	Chr4	15421465	15461465	15441465
KEE09	Chr5	4780379	4820379	4800379
KEE10	Chr5	10311725	10351725	10331725

Table S2 Transgene copy number analyzed by droplet digital PCR (ddPCR). The transgene copy number was assessed using the *NPTII* (*KAN*) gene relative to two endogenous single copy loci (*FIE* (AT3G20740) and *LYS* (AT5G62150)). The final copy number was determined by the mean of *KAN/FIE* and *KAN/LYS* ratios.

Parental	F1	***KAN*** **rep1** (copies/µl)	***KAN*** **rep2** (copies/µl)	***FIE*** **rep1** (copies/µl)	***FIE*** **rep2** (copies/µl)	***LYS*** **rep1** (copies/µl)	***LYS*** **rep2** (copies/µl)	***KAN/FIE***	***KAN/LYS***	**Copy number**
SG260	SG261	55.9	62.9	59.1	53.8	61.1	54.3	1.1	1	1
SG260	SG368	36.2	35.1	38	39.3	39.8	36.4	0.9	0.9	1
SG260	SG371	87	82.8	96.6	107.7	91.8	108.7	0.8	0.8	1
SG292	SG335	52.3	49.5	50.5	50.3	54	49.1	1	1	1
SG292	SG337	132	144.7	145	148	145.6	159	0.9	0.9	1
SG292	SG369	170	146	169	168	177	182	0.9	0.9	1
SG298	SG350	298	302	324	319	337	309	0.9	0.9	1
SG298	SG361	168	161	160	171	184	175	1	0.9	1
SG298	SG362	61.9	61.7	51.1	42.8	43.7	47.6	1.3	1.4	1
SG307	SG342	304	284	90.1	84	82.9	78.9	3.4	3.6	4
SG307	SG355	346	370	139	119	85.6	128	2.8	3.4	3
SG307	SG356	408	494	144.7	176	159	206	2.8	2.5	3
SG310	SG340	127.7	115	134	133	135	126	0.9	0.9	1
SG310	SG358	37.9	40.7	38.7	41.3	40.4	42.5	1	0.9	1
SG314	SG346	628	576	215	215	217	258	2.8	2.5	3
SG314	SG354	562	499	166	187	183	196	3	2.8	3
SG314	SG357	355	361	116.9	125.8	133.3	129.3	3	2.7	3
SG330	SG352	472	484	94	103.2	107.8	103.4	4.8	4.5	5
SG330	SG353	926	843	134.8	139	139	153	6.5	6.1	6
SG330	SG359	1334	1390	275	246	273	281	5.2	4.9	5
SG333	SG366	70.8	60.9	61.9	69.5	76.8	76.2	1	0.9	1
SG333	SG367	50.2	51.6	55.2	53.4	63.8	63.5	0.9	0.8	1
SG333	SG373	35.4	36.7	43.8	41	42.8	44	0.9	0.8	1
SG339	SG339B	0.2	0.07	164	168	171	60.2	0	0	0
SG339	SG339C	0.07	0.15	105.9	105	112	111.8	0	0	0

Table S3. Kanamycin resistance score. Viability phenotype (scale 0 (dead) to 10 (no effect of Kanamycin)) was scored double blindly based on images acquired from 14-day-old seedlings grown on kanamycin containing medium.

Parental	F1	score 1	score 2	mean score
SG260	SG261	7	7	7
SG260	SG368	7	7	7
SG260	SG371	7	7	7
SG292	SG335	5	6	5.5
SG292	SG337	8	8	8
SG292	SG369	7	8	7.5
SG298	SG350	7	7	7
SG298	SG361	8	7	7.5
SG298	SG362	7	7	7
SG307	SG342	5	6	5.5
SG307	SG355	5	5	5
SG307	SG356	4	5	4.5
SG310	SG340	9	8	8.5
SG310	SG358	9	8	8.5
SG314	SG346	0	0	0
SG314	SG354	0	0	0
SG314	SG357	0	0	0
SG330	SG352	3	3	3
SG330	SG353	3	4	3.5
SG330	SG359	0	0	0
SG333	SG366	8	8	8
SG333	SG367	8	8	8
SG333	SG373	9	7	8
SG339	SG339A	0	0	0
SG339	SG339B	0	0	0
SG339	SG339C	0	0	0

Table S4. Seedling viability of 99 transgenic lines.

Line	Transgene insertion site (Chr1)	Genomic bin (300kb)	Viability score
SG535	163419	1	1
SG530	389812	300001	1.5
SG521	880496	600001	1
SG564	982506	900001	1
SG519	1431190	1200001	2
SG524	1569192	1500001	4
SG346	2085299	1800001	1
SG539	2249133	2100001	4.5
SG489	2498836	2400001	10
SG341	2952334	2700001	6
SG555	3064124	3000001	1.5
SG511	3442327	3300001	2
SG561	4084162	3900001	3
SG554	4286204	4200001	4
SG505	4694372	4500001	1.5
SG568	4927011	4800001	1.5
SG547	5138334	5100001	7
SG498	5578431	5400001	6
SG315	6009988	6000001	1.5
SG546	6528984	6300001	1
SG531	6765667	6600001	3.5
SG309	7042672	6900001	3
SG335	7458618	7200001	8
SG556	7232006	7200001	1
SG548	7527079	7500001	2
SG313	8002649	7800001	3
SG518	8317396	8100001	2.5
SG549	8584039	8400001	7
SG516	8951202	8700001	6
SG558	9076048	9000001	3
SG553	9481532	9300001	2
SG520	9611103	9600001	2
SG565	9907202	9900001	4.5
SG502	10407379	10200001	2.5
SG522	10790022	10500001	2
SG311	11012274	10800001	2
SG506	11374893	11100001	2
SG513	11575320	11400001	2
SG570	11993298	11700001	2
SG503	12087580	12000001	1
SG544	12398435	12300001	1
SG494	12842158	12600001	1
SG340	12964622	12900001	8
SG532	13277274	13200001	2
SG534	13669441	13500001	7
SG304	14056029	13800001	1
SG537	14313049	14100001	1
SG508	14543277	14400001	1.5
SG571	15179141	15000001	8
SG499	15485350	15300001	8.5
SG491	15849233	15600001	2
SG316	15951543	15900001	2
SG496	16266641	16200001	1
SG509	16646732	16500001	1
SG373	17053813	16800001	9
SG487	17165127	17100001	5
SG512	17603504	17400001	7.5
SG351	17995869	17700001	8
SG541	18284335	18000001	1
SG540	18473908	18300001	1
SG517	18876837	18600001	1
SG567	19136251	18900001	2
SG507	19201789	19200001	2
SG306	20068163	19800001	1
SG488	20286882	20100001	2
SG528	20433912	20400001	8
SG526	20715563	20700001	1
SG308	21043366	21000001	6.5
SG504	21408623	21300001	1
SG533	21839858	21600001	6
SG305	22043881	21900001	7
SG563	22043829	21900001	1.5
SG543	22271226	22200001	1
SG350	22625361	22500001	9.5
SG368	22642823	22500001	8
SG566	22911205	22800001	1
SG486	23230956	23100001	1.5
SG523	23577333	23400001	2
SG569	23795050	23700001	1.5
SG500	24054143	24000001	2
SG545	24489780	24300001	3
SG527	24861876	24600001	5.5
SG536	25198182	24900001	2
SG515	25474140	25200001	1
SG551	25746736	25500001	7
SG550	26378794	26100001	5.5
SG352	26951796	26700001	3.5
SG497	27437928	27300001	8
SG538	27752400	27600001	6.5
SG495	28151924	27900001	1
SG525	28327886	28200001	3
SG559	28705527	28500001	2
SG303	29044174	28800001	7
SG560	28960387	28800001	1
SG510	29148256	29100001	1.5
SG514	29610494	29400001	3.5
SG529	29976725	29700001	3
SG318	30038603	30000001	1.5
SG493	30405432	30300001	5

Table S5. Methylation analysis by Sanger sequencing after bisulfite treatment. Target: Chloroplast DNA. Chloroplast DNA is not methylated and serves to assess bisulfite conversion efficiency. Hence, the column "Unmethylated (percent)" indicates bisulfite conversion efficiency.

Parental	F1	Context	Methylated (percent)	Unmethylated (percent)	Number of mC	Number of C	Total Cytosines	Clones analyzed
SG292	SG335	All	1.7%	98.3%	13	732	745	25
SG292	SG335	CG	1.4%	98.6%	1	72	73	25
SG292	SG335	CHG	1.3%	98.7%	2	147	149	25
SG292	SG335	CHH	1.9%	98.1%	10	513	523	25
SG292	SG369	All	6.2%	93.8%	11	166	177	6
SG292	SG369	CG	6.3%	93.8%	1	15	16	6
SG292	SG369	CHG	5.6%	94.4%	2	34	36	6
SG292	SG369	CHH	6.4%	93.6%	8	117	125	6
SG298	SG350	All	2.6%	97.5%	13	497	510	17
SG298	SG350	CG	0.0%	100.0%	0	51	51	17
SG298	SG350	CHG	4.9%	95.1%	5	97	102	17
SG298	SG350	CHH	2.2%	97.8%	8	349	357	17
SG298	SG362	All	4.7%	95.3%	21	427	448	15
SG298	SG362	CG	2.3%	97.7%	1	43	44	15
SG298	SG362	CHG	4.5%	95.5%	4	85	89	15
SG298	SG362	CHH	5.1%	94.9%	16	299	315	15
SG307	SG355	All	0.7%	99.3%	3	417	420	14
SG307	SG355	CG	0.0%	100.0%	0	42	42	14
SG307	SG355	CHG	0.0%	100.0%	0	84	84	14
SG307	SG355	CHH	1.0%	99.0%	3	291	294	14
SG307	SG356	All	2.8%	97.2%	11	378	389	13
SG307	SG356	CG	2.6%	97.4%	1	38	39	13
SG307	SG356	CHG	2.6%	97.4%	2	76	78	13
SG307	SG356	CHH	2.9%	97.1%	8	264	272	13
SG310	SG358	All	1.1%	98.9%	3	267	270	9
SG310	SG358	CG	0.0%	100.0%	0	27	27	9
SG310	SG358	CHG	0.0%	100.0%	0	54	54	9
SG310	SG358	CHH	1.6%	98.4%	3	186	189	9
SG314	SG346	All	1.2%	98.8%	4	326	330	11
SG314	SG346	CG	0.0%	100.0%	0	33	33	11
SG314	SG346	CHG	1.5%	98.5%	1	65	66	11
SG314	SG346	CHH	1.3%	98.7%	3	228	231	11
SG314	SG357	All	4.5%	95.5%	20	427	447	15
SG314	SG357	CG	4.7%	95.3%	2	41	43	15
SG314	SG357	CHG	4.4%	95.6%	4	86	90	15
SG314	SG357	CHH	4.5%	95.5%	14	300	314	15
SG330	SG349	All	3.4%	96.7%	14	404	418	14
SG330	SG349	CG	4.9%	95.1%	2	39	41	14
SG330	SG349	CHG	1.2%	98.8%	1	82	83	14
SG330	SG349	CHH	3.7%	96.3%	11	283	294	14
SG330	SG353	All	2.1%	97.9%	5	235	240	8
SG330	SG353	CG	4.2%	95.8%	1	23	24	8
SG330	SG353	CHG	4.2%	95.8%	2	46	48	8
SG330	SG353	CHH	1.2%	98.8%	2	166	168	8
SG330	SG359	All	2.4%	97.6%	13	520	533	18
SG330	SG359	CG	1.9%	98.1%	1	51	52	18
SG330	SG359	CHG	0.0%	100.0%	0	107	107	18
SG330	SG359	CHH	3.2%	96.8%	12	362	374	18

Table S6. Methylation analysis by Sanger sequencing after bisulfite sequencing. Target: nopaline synthase promoter (*nosP*).

Parental	F1	Context	Methylated (percent)	Unmethylated (percent)	Number of mC	Number of C	Total Cytosines	Clones analyzed
SG292	SG335	All	7.7%	92.3%	93	1111	1204	17
SG292	SG335	CG	14.7%	85.3%	50	289	339	17
SG292	SG335	CHG	8.4%	91.6%	20	218	238	17
SG292	SG335	CHH	3.7%	96.3%	23	604	627	17
SG292	SG337	All	5.9%	94.1%	67	1064	1131	16
SG292	SG337	CG	14.4%	85.6%	46	274	320	16
SG292	SG337	CHG	4.0%	96.0%	9	214	223	16
SG292	SG337	CHH	2.0%	98.0%	12	576	588	16
SG292	SG369	All	11.2%	88.8%	71	562	633	9
SG292	SG369	CG	21.1%	78.9%	38	142	180	9
SG292	SG369	CHG	9.6%	90.4%	12	113	125	9
SG292	SG369	CHH	6.4%	93.6%	21	307	328	9
SG298	SG350	All	10.2%	89.9%	172	1522	1694	24
SG298	SG350	CG	23.9%	76.1%	114	363	477	24
SG298	SG350	CHG	7.8%	92.2%	26	307	333	24
SG298	SG350	CHH	3.6%	96.4%	32	852	884	24
SG298	SG362	All	10.8%	89.2%	107	884	991	15
SG298	SG362	CG	24.2%	75.8%	69	216	285	15
SG298	SG362	CHG	5.6%	94.4%	11	184	195	15
SG298	SG362	CHH	5.3%	94.7%	27	484	511	15
SG307	SG342	All	8.9%	91.1%	77	785	862	13
SG307	SG342	CG	18.6%	81.4%	46	201	247	13
SG307	SG342	CHG	2.4%	97.6%	4	164	168	13
SG307	SG342	CHH	6.0%	94.0%	27	420	447	13
SG307	SG355	All	4.4%	95.6%	84	1811	1895	27
SG307	SG355	CG	8.6%	91.4%	46	491	537	27
SG307	SG355	CHG	3.2%	96.8%	12	361	373	27
SG307	SG355	CHH	2.6%	97.4%	26	959	985	27
SG307	SG356	All	10.7%	89.3%	187	1556	1743	28
SG307	SG356	CG	23.9%	76.1%	119	378	497	28
SG307	SG356	CHG	7.0%	93.0%	24	317	341	28
SG307	SG356	CHH	4.9%	95.1%	44	861	905	28
SG310	SG340	All	8.2%	91.8%	97	1082	1179	17
SG310	SG340	CG	14.5%	85.5%	48	284	332	17
SG310	SG340	CHG	9.9%	90.1%	23	210	233	17
SG310	SG340	CHH	4.2%	95.8%	26	588	614	17
SG310	SG358	All	3.4%	96.6%	41	1163	1204	17
SG310	SG358	CG	6.8%	93.2%	23	315	338	17
SG310	SG358	CHG	2.5%	97.5%	6	232	238	17
SG310	SG358	CHH	1.9%	98.1%	12	616	628	17
SG314	SG346	All	23.4%	76.6%	432	1414	1846	26
SG314	SG346	CG	52.3%	47.7%	272	248	520	26
SG314	SG346	CHG	24.7%	75.3%	90	274	364	26
SG314	SG346	CHH	7.3%	92.7%	70	892	962	26
SG314	SG354	All	24.8%	75.2%	176	534	710	10
SG314	SG354	CG	55.0%	45.0%	110	90	200	10
SG314	SG354	CHG	27.1%	72.9%	38	102	140	10
SG314	SG354	CHH	7.6%	92.4%	28	342	370	10
SG314	SG357	All	24.0%	76.0%	419	1329	1748	25
SG314	SG357	CG	56.7%	43.3%	281	215	496	25
SG314	SG357	CHG	22.4%	77.6%	77	267	344	25
SG314	SG357	CHH	6.7%	93.3%	61	847	908	25
SG330	SG349	All	12.2%	87.8%	285	2048	2333	36
SG330	SG349	CG	26.9%	73.1%	177	480	657	36
SG330	SG349	CHG	9.9%	90.1%	46	418	464	36
SG330	SG349	CHH	5.1%	94.9%	62	1150	1212	36
SG330	SG353	All	7.0%	93.1%	38	509	547	8
SG330	SG353	CG	16.6%	83.4%	26	131	157	8
SG330	SG353	CHG	4.6%	95.4%	5	103	108	8
SG330	SG353	CHH	2.5%	97.5%	7	275	282	8
SG330	SG359	All	13.2%	86.8%	280	1840	2120	32
SG330	SG359	CG	30.5%	69.5%	183	417	600	32
SG330	SG359	CHG	10.2%	89.8%	43	377	420	32
SG330	SG359	CHH	4.9%	95.1%	54	1046	1100	32

Table S7. Viability analysis by digital image analysis. Images acquired from 14-day-old seedlings were used to determine total area covered by seedling tissue and mean grey value of the green channel using the ImageJ image analysis software.

F1	Area	Mean value green channel	Area x Mean	Max	Maternal line	Paternal line	Maternal phenotype	Paternal phenotype	Progeny class
SG381	2715218	203.0	551267995.3	255	SG350	SG369	active	active	aa
SG383	2317627	194.7	451177083.3	253	SG358	SG369	active	active	aa
SG387	2516087	197.9	497888327.7	255	SG369	SG350	active	active	aa
SG389	1875489	195.7	366971306.2	255	SG358	SG350	active	active	aa
SG399	1876518	196.6	368902797.1	252	SG369	SG358	active	active	aa
SG400	2147235	200.0	429358963.4	251	SG350	SG358	active	active	aa
SG417	2443513	195.4	477430674.5	255	SG369	SG369	active	active	aa
SG418	2652069	199.8	530000077.2	251	SG350	SG350	active	active	aa
SG420	2590169	195.7	506831319.1	248	SG358	SG358	active	active	aa
SG393	2452259	191.8	470409487.2	253	SG369	SG356	active	silenced	as
SG394	2090897	190.9	399194055.2	251	SG350	SG356	active	silenced	as
SG395	2728205	200.3	546530394.8	255	SG358	SG356	active	silenced	as
SG405	2817386	194.5	548052011.7	246	SG369	SG346	active	silenced	as
SG406	2247877	200.1	449692289.6	255	SG350	SG346	active	silenced	as
SG408	2183235	196.9	429876788.3	241	SG358	SG346	active	silenced	as
SG411	2356989	197.6	465854161.9	255	SG369	SG359	active	silenced	as
SG412	2291818	197.5	452654681.4	255	SG350	SG359	active	silenced	as
SG414	2719761	196.0	532997002.7	253	SG358	SG359	active	silenced	as
SG374	2724989	197.9	539144523.6	255	SG369	SG339	active	WT	aw
SG375	2441905	194.9	475829608.3	255	SG350	SG339	active	WT	aw
SG377	2472109	197.2	487517199.6	253	SG358	SG339	active	WT	aw
SG382	2066800	197.3	407856111.6	247	SG356	SG369	silenced	active	sa
SG384	2208681	200.9	443715178.2	255	SG346	SG369	silenced	active	sa
SG385	2539552	193.9	492436909.7	250	SG359	SG369	silenced	active	sa
SG388	2637839	196.5	518235125.6	254	SG356	SG350	silenced	active	sa
SG390	1873755	195.7	366647009.6	255	SG346	SG350	silenced	active	sa
SG391	2262987	194.8	440857023.4	255	SG359	SG350	silenced	active	sa
SG401	2030258	196.6	399171055.6	247	SG356	SG358	silenced	active	sa
SG402	2415199	201.7	487155299.1	255	SG346	SG358	silenced	active	sa
SG403	2382035	197.1	469396671	253	SG359	SG358	silenced	active	sa
SG396	949500	189.7	180085018.5	255	SG346	SG356	silenced	silenced	ss
SG397	1183023	187.7	222025024.5	255	SG359	SG356	silenced	silenced	ss
SG407	929527	189.3	175945518.2	255	SG356	SG346	silenced	silenced	ss
SG409	876358	189.5	166028652.2	255	SG359	SG346	silenced	silenced	ss
SG413	1315324	182.7	240316271.4	255	SG356	SG359	silenced	silenced	ss
SG415	1037578	184.2	191099040.9	255	SG346	SG359	silenced	silenced	ss
SG419	1033947	188.8	195174039.4	254	SG356	SG356	silenced	silenced	ss
SG421	739776	187.9	139011308.2	255	SG346	SG346	silenced	silenced	ss
SG422	764893	188.2	143949038.1	255	SG359	SG359	silenced	silenced	ss
SG376	1052762	186.6	196416964.6	255	SG356	SG339	silenced	WT	sw
SG378	1313899	184.4	242326334.3	255	SG346	SG339	silenced	WT	sw
SG379	899555	186.2	167477350.8	254	SG359	SG339	silenced	WT	sw
SG380	2699148	196.9	531373169.3	247	SG339	SG369	WT	active	wa
SG386	2469660	195.1	481914634.4	255	SG339	SG350	WT	active	wa
SG398	2588310	198.8	514649207.2	255	SG339	SG358	WT	active	wa
SG392	1286285	187.4	241069103.3	255	SG339	SG356	WT	silenced	ws
SG404	656335	185.6	121838747.7	255	SG339	SG346	WT	silenced	ws
SG410	950428	189.2	179803869.9	255	SG339	SG359	WT	silenced	ws
SG416	1240365	184.7	229076810	255	SG339	SG339	WT	WT	ww

Table S8. Phenotypic segregation analysis of F2 seedling populations. Seeds from four F1 self-crossed siblings were pooled and plated on media containing kanamycin. Kanamycin sensitivity was assessed in 14-day-old seedlings. *: scores were taken over from pooled data of **Table S9**. r: resistant, s: sensitive, WT: wild-type

Selfed F1	Sensitive offspring	Resistant offspring	Total analyzed	Not germinated	Mother	Father	Ancestral maternal genotype	Ancestral paternal genotype	Ancestral phenotype (m/p)	Replicate
SG399	3	49	52	0	SG369	SG358	SG292	SG310	r/r	Rep1
SG417	0	52	52	0	SG369	SG369	SG292	SG292	r/r	Rep1
SG400	1	49	50	2	SG350	SG358	SG298	SG310	r/r	Rep1
SG381	2	49	51	1	SG350	SG369	SG298	SG292	r/r	Rep1
SG418	1	47	48	4	SG350	SG350	SG298	SG298	r/r	Rep1
SG383	1	49	50	2	SG358	SG369	SG310	SG292	r/r	Rep1
SG420	1	51	52	0	SG358	SG358	SG310	SG310	r/r	Rep1
SG405	28	19	47	5	SG369	SG346	SG292	SG314	r/s	Rep1
SG393	11	37	48	4	SG369	SG356	SG292	SG307	r/s	Rep1
SG411	24	28	52	0	SG369	SG359	SG292	SG330	r/s	Rep1
SG406	22	29	51	1	SG350	SG346	SG298	SG314	r/s	Rep1
SG394	17	29	46	6	SG350	SG356	SG298	SG307	r/s	Rep1
SG412*	129	65	194	14	SG350	SG359	SG298	SG330	r/s	Rep1
SG408	8	40	48	4	SG358	SG346	SG310	SG314	r/s	Rep1
SG374	18	28	46	6	SG369	SG339	SG292	SG339	r/WT	Rep1
SG375	13	36	49	3	SG350	SG339	SG298	SG339	r/WT	Rep1
SG377	14	38	52	0	SG358	SG339	SG310	SG339	r/WT	Rep1
SG388	9	40	49	3	SG356	SG350	SG307	SG298	s/r	Rep1
SG401*	35	112	147	7	SG356	SG358	SG307	SG310	s/r	Rep1
SG382	8	43	51	1	SG356	SG369	SG307	SG292	s/r	Rep1
SG390	19	29	48	4	SG346	SG350	SG314	SG298	s/r	Rep1
SG402	20	32	52	0	SG346	SG358	SG314	SG310	s/r	Rep1
SG384	13	37	50	2	SG346	SG369	SG314	SG292	s/r	Rep1
SG391*	164	33	197	11	SG359	SG350	SG330	SG298	s/r	Rep1
SG403*	179	16	195	13	SG359	SG358	SG330	SG310	s/r	Rep1
SG385	24	28	52	0	SG359	SG369	SG330	SG292	s/r	Rep1
SG407	36	11	47	5	SG356	SG346	SG307	SG314	s/s	Rep1
SG419	46	5	51	1	SG356	SG356	SG307	SG307	s/s	Rep1
SG396	23	24	47	5	SG346	SG356	SG314	SG307	s/s	Rep1
SG415	50	0	50	2	SG346	SG359	SG314	SG330	s/s	Rep1
SG421	51	0	51	1	SG346	SG346	SG314	SG314	s/s	Rep1
SG409	49	0	49	3	SG359	SG346	SG330	SG314	s/s	Rep1
SG397	50	1	51	1	SG359	SG356	SG330	SG307	s/s	Rep1
SG422	49	0	49	3	SG359	SG359	SG330	SG330	s/s	Rep1
SG416	52	0	52	0	SG339	SG339	SG339	SG339	s/s	Rep1
SG376	48	0	48	4	SG356	SG339	SG307	SG339	s/WT	Rep1
SG378	52	0	52	0	SG346	SG339	SG314	SG339	s/WT	Rep1
SG379	49	0	49	3	SG359	SG339	SG330	SG339	s/WT	Rep1
SG386	17	34	51	1	SG339	SG350	SG339	SG298	WT/r	Rep1
SG398	14	36	50	2	SG339	SG358	SG339	SG310	WT/r	Rep1
SG380	7	43	50	2	SG339	SG369	SG339	SG292	WT/r	Rep1
SG404	52	0	52	0	SG339	SG346	SG339	SG314	WT/s	Rep1
SG392	47	0	47	5	SG339	SG356	SG339	SG307	WT/s	Rep1
SG410	52	0	52	0	SG339	SG359	SG339	SG330	WT/s	Rep1
SG436	1	47	48	4	SG369	SG350	SG292	SG298	r/r	Rep2
SG448	3	47	50	2	SG369	SG358	SG292	SG310	r/r	Rep2
SG466	0	49	49	3	SG369	SG369	SG292	SG292	r/r	Rep2
SG449	1	50	51	1	SG350	SG358	SG298	SG310	r/r	Rep2
SG467	51	0	51	1	SG350	SG350	SG298	SG298	r/r	Rep2
SG469	1	47	48	4	SG358	SG358	SG310	SG310	r/r	Rep2
SG454*	37	114	151	5	SG369	SG346	SG292	SG314	r/s	Rep2
SG442	9	40	49	3	SG369	SG356	SG292	SG307	r/s	Rep2
SG460	17	34	51	1	SG369	SG359	SG292	SG330	r/s	Rep2
SG455	19	30	49	3	SG350	SG346	SG298	SG314	r/s	Rep2
SG443	7	44	51	1	SG350	SG356	SG298	SG307	r/s	Rep2
SG461	31	21	52	0	SG350	SG359	SG298	SG330	r/s	Rep2
SG457	8	40	48	4	SG358	SG346	SG310	SG314	r/s	Rep2
SG444	12	37	49	3	SG358	SG356	SG310	SG307	r/s	Rep2
SG463*	153	33	186	22	SG358	SG359	SG310	SG330	r/s	Rep2
SG423	8	44	52	0	SG369	SG339	SG292	SG339	r/WT	Rep2
SG424	6	44	50	2	SG350	SG339	SG298	SG339	r/WT	Rep2
SG426	11	41	52	0	SG358	SG339	SG310	SG339	r/WT	Rep2
SG437	9	38	47	5	SG356	SG350	SG307	SG298	s/r	Rep2
SG450	8	43	51	1	SG356	SG358	SG307	SG310	s/r	Rep2
SG431	13	39	52	0	SG356	SG369	SG307	SG292	s/r	Rep2
SG439	18	30	48	4	SG346	SG350	SG314	SG298	s/r	Rep2
SG451*	81	64	145	11	SG346	SG358	SG314	SG310	s/r	Rep2
SG433	26	23	49	3	SG346	SG369	SG314	SG292	s/r	Rep2
SG440*	165	24	189	19	SG359	SG350	SG330	SG298	s/r	Rep2
SG452	38	14	52	0	SG359	SG358	SG330	SG310	s/r	Rep2
SG434	15	35	50	2	SG359	SG369	SG330	SG292	s/r	Rep2
SG456	49	0	49	3	SG356	SG346	SG307	SG314	s/s	Rep2
SG462	51	0	51	1	SG356	SG359	SG307	SG330	s/s	Rep2
SG468	3	49	52	0	SG356	SG356	SG307	SG307	s/s	Rep2
SG445	49	0	49	3	SG346	SG356	SG314	SG307	s/s	Rep2
SG464	47	0	47	5	SG346	SG359	SG314	SG330	s/s	Rep2
SG470	50	0	50	2	SG346	SG346	SG314	SG314	s/s	Rep2
SG458	49	2	51	1	SG359	SG346	SG330	SG314	s/s	Rep2
SG446	52	0	52	0	SG359	SG356	SG330	SG307	s/s	Rep2
SG471	51	0	51	1	SG359	SG359	SG330	SG330	s/s	Rep2
SG465	52	0	52	0	SG339	SG339	SG339	SG339	s/s	Rep2
SG425	48	4	52	0	SG356	SG339	SG307	SG339	s/WT	Rep2
SG427	48	4	52	0	SG346	SG339	SG314	SG339	s/WT	Rep2
SG428	52	0	52	0	SG359	SG339	SG330	SG339	s/WT	Rep2
SG435	16	34	50	2	SG339	SG350	SG339	SG298	WT/r	Rep2
SG447	12	40	52	0	SG339	SG358	SG339	SG310	WT/r	Rep2
SG429	11	41	52	0	SG339	SG369	SG339	SG292	WT/r	Rep2
SG453	50	0	50	2	SG339	SG346	SG339	SG314	WT/s	Rep2
SG441	47	0	47	5	SG339	SG356	SG339	SG307	WT/s	Rep2
SG459	52	0	52	0	SG339	SG359	SG339	SG330	WT/s	Rep2

Table S9. Phenotypic segregation analysis of F2 seedling populations. Seeds from a subset of individual F1 self-crossed plants were plated on media containing kanamycin. Kanamycin sensitivity was visually assessed in 14-day-old seedlings. r: resistant, s: sensitive.

**Selfed F1 individual**	**Sensitive offspring**	**Resistant Offspring**	**Total analyzed**	**Not germinated**	**Mother**	**Father**	**Ancestral maternal genotype**	**Ancestral paternal genotype**	**Replicate**	**Ancestral phenotype (m/p)**
SG391-1	50	1	51	1	SG359	SG350	SG330	SG298	Rep1	s/r
SG391-2	20	31	51	1	SG359	SG350	SG330	SG298	Rep1	s/r
SG391-3	48	0	48	4	SG359	SG350	SG330	SG298	Rep1	s/r
SG391-4	46	1	47	5	SG359	SG350	SG330	SG298	Rep1	s/r
SG401-1	11	40	51	1	SG356	SG358	SG307	SG310	Rep1	s/r
SG401-2	15	30	45	5	SG356	SG358	SG307	SG310	Rep1	s/r
SG401-3	9	42	51	1	SG356	SG358	SG307	SG310	Rep1	s/r
SG403-1	49	2	51	1	SG359	SG358	SG330	SG310	Rep1	s/r
SG403-2	36	11	47	5	SG359	SG358	SG330	SG310	Rep1	s/r
SG403-3	50	0	50	2	SG359	SG358	SG330	SG310	Rep1	s/r
SG403-4	44	3	47	5	SG359	SG358	SG330	SG310	Rep1	s/r
SG412-1	21	25	46	6	SG350	SG359	SG298	SG330	Rep1	r/s
SG412-2	44	5	49	3	SG350	SG359	SG298	SG330	Rep1	r/s
SG412-3	33	16	49	3	SG350	SG359	SG298	SG330	Rep1	r/s
SG412-4	31	19	50	2	SG350	SG359	SG298	SG330	Rep1	r/s
SG440-1	41	1	42	10	SG359	SG350	SG330	SG298	Rep2	s/r
SG440-2	49	0	49	3	SG359	SG350	SG330	SG298	Rep2	s/r
SG440-3	26	22	48	4	SG359	SG350	SG330	SG298	Rep2	s/r
SG440-4	49	1	50	2	SG359	SG350	SG330	SG298	Rep2	s/r
SG451-1	23	25	48	4	SG346	SG358	SG314	SG310	Rep2	s/r
SG451-2	39	10	49	3	SG346	SG358	SG314	SG310	Rep2	s/r
SG451-4	19	29	48	4	SG346	SG358	SG314	SG310	Rep2	s/r
SG452-1	40	9	49	3	SG359	SG358	SG330	SG310	Rep2	s/r
SG452-2	35	14	49	3	SG359	SG358	SG330	SG310	Rep2	s/r
SG452-3	41	7	48	4	SG359	SG358	SG330	SG310	Rep2	s/r
SG452-4	26	24	50	2	SG359	SG358	SG330	SG310	Rep2	s/r
SG454-1	12	39	51	1	SG369	SG346	SG292	SG314	Rep2	r/s
SG454-2	9	39	48	4	SG369	SG346	SG292	SG314	Rep2	r/s
SG454-3	16	36	52	0	SG369	SG346	SG292	SG314	Rep2	r/s
SG461-1	41	8	49	3	SG350	SG359	SG298	SG330	Rep2	r/s
SG461-2	50	0	50	2	SG350	SG359	SG298	SG330	Rep2	r/s
SG461-3	48	1	49	3	SG350	SG359	SG298	SG330	Rep2	r/s
SG463-1	38	13	51	1	SG358	SG359	SG310	SG330	Rep2	r/s
SG463-2	41	8	49	3	SG358	SG359	SG310	SG330	Rep2	r/s
SG463-3	34	10	44	8	SG358	SG359	SG310	SG330	Rep2	r/s
SG463-4	40	2	42	10	SG358	SG359	SG310	SG330	Rep2	r/s

Table S10. PCR-based Genotyping of selected F2 seedling populations. PCR was performed using primers either spanning the insertion site (wild-type) or using a combination of a T-DNA specific primer and an endogenous primer (see also **Table S13**). Statistical analysis was performed using Chi-Square tests, followed by adjusting the p-values for multiple testing (Benjamini-Hochberg).

T-DNA	homozygous	heterozygous	wild type	F1 parent	ChiSq *P*	FDR
SG310	11	17	11	SG463_1	0.73	0.97
SG310	10	19	11	SG463_2	0.93	0.97
SG310	12	29	10	SG463_3	0.57	0.96
SG310	20	18	12	SG463_4	0.04	0.37
SG310	11	26	14	SG452_1	0.83	0.97
SG310	11	23	16	SG452_2	0.52	0.92
SG310	13	28	11	SG452_3	0.79	0.97
SG310	10	31	10	SG452_4	0.31	0.80
SG298	9	34	8	SG440_1	0.06	0.37
SG298	18	25	9	SG440_2	0.20	0.65
SG298	10	33	9	SG440_3	0.15	0.61
SG298	11	32	8	SG440_4	0.16	0.61
SG298	14	23	14	SG412_1	0.78	0.97
SG298	13	25	14	SG412_2	0.94	0.97
SG298	10	30	10	SG412_3	0.37	0.80
SG298	14	31	6	SG412_4	0.09	0.46
SG330	10	20	6	SG463_1	0.51	0.92
SG330	7	15	8	SG463_2	0.97	0.97
SG330	10	16	10	SG463_3	0.80	0.97
SG330	13	22	9	SG463_4	0.70	0.97
SG330	7	21	12	SG452_1	0.51	0.92
SG330	8	20	4	SG452_2	0.22	0.65
SG330	16	20	5	SG452_3	0.05	0.37
SG330	4	32	12	SG452_4	0.02	0.37
SG330	10	26	13	SG440_1	0.76	0.97
SG330	14	30	7	SG440_2	0.17	0.61
SG330	11	31	10	SG440_3	0.38	0.80
SG330	12	28	12	SG440_4	0.86	0.97
SG330	12	22	13	SG412_1	0.89	0.97
SG330	20	18	12	SG412_2	0.04	0.37
SG330	12	27	12	SG412_3	0.92	0.97
SG330	9	30	11	SG412_4	0.34	0.80

Table S11. Enrichment of *VANDAL6* and *ATLANTYS3* transposable elements in canonical *KEEs* (10 *KEEs*) and ectopic *KEEs* (10 ectopic *KEEs* in *ddm1* mutants (Feng et al., 2014)). Monte-Carlo based statistical testing revealed significant enrichment ($P < 0.0001$). Ectopic and canonical genomic *KEE* regions were defined as a 40 kb genomic region centered around the *KNOT* interaction maxima.

TE family	canonical *KEEs*	ectopic *KEEs*	Total in genome	% within all *KEEs*
VANDAL6	19	19	89	43
ATLANTYS3	18	12	142	21

Table S12. 4C amplification primers.

Viewpoint	4C primer 1	4C primer 2	1° RS	2° RS	#cycles	annealing
SG260	TCGAAAGCAACAGACTTTGGA	CAGTCCAAACAACTCTCGGA	HindIII	DpnII	26	63°C
SG292	TCTCCATGTTCGAACAACGT	TGAAAGAGAGAATCCAAGCAGAG	HindIII	DpnII	26	63°C
SG298	TCCGGCTTTCTCGTACTTGT	TCTTTGTTCTTTGCGATCCGA	HindIII	TaqαI	26	64°C
SG307	TCCCCTGTAAGCACAACAGA	GTCTTCTGATGTGGCTGCCA	HindIII	NlaIII	26	63°C
SG310	TCTTGCTGTCAGGTCAAGCT	TCGACATGCTACATGATAGAACT	HindIII	DpnII	26	60°C
SG314	ACGTCCCTTACCATCACACC	TGTTGTTGCTTGTAACCATTCTT	HindIII	DpnII	26	62°C
SG330	TCTGAAGCATCTCAATCTCTTGC	AGTGCAAATGTTAGGGAGAGTGA	HindIII	DpnII	26	65°C
SG333	TTTCTGCTCTTGCTTCTCTGA	GGTGTGAGACTTAACGCAACA	HindIII	TaqαI	26	65°C
KEE6	TCTCTGTTCTCAAAAGAGCAAAC	TGGCCGTTATTCAATTTCCCG	HindIII	DpnII	29	65°C

Table S13. Genotyping primers. RP and LP primers bind to genomic DNA sequence, whereas the RB primer binds to the right border of the *T-DNA* transgene.

Parental	RP	LP	RB
SG260	TCCAACACAGAAACTTGGTCC	ACTCTCTTGGACTTGCATTGC	ATTTTGCCGATTTCGGAAC
SG292	AGATACAGTTTTGTCGGGTCG	TCCATGGAATAAGAGAAAAGAGC	ATTTTGCCGATTTCGGAAC
SG298	ATGTGAGCTAGGCCTTAAGCC	TTTGAAACATCACAGCGAGTG	ATTTTGCCGATTTCGGAAC
SG307	CCTTTGGTTATGCGAAATGAG	CAAGAAACAAAGCACTGCAAAC	ATTTTGCCGATTTCGGAAC
SG310	TCTTGAACTCCATGTTCCAGG	TTTAACTTCTTGTCGCGAAGG	ATTTTGCCGATTTCGGAAC
SG314	AAACCACATTGAGATTGCTGG	GAACTTGATGATTGCTCAGGG	ATTTTGCCGATTTCGGAAC
SG330	CCTCGTCTTCGACATAACTGG	AACTTACCAATCCCATCGACC	ATTTTGCCGATTTCGGAAC
SG333	CGGATCAGAAACTCTTGCTTG	GAGAGAACAAGCGGTGTTGAC	ATTTTGCCGATTTCGGAAC

Table S14. Droplet digital PCR primer and probe sequences.

Target	Primer 1	Primer 2	MGB probe	Probe labeling
FIE	TAGCAAAGCGGTAAATATCACG	TGAAGTTCTAAGTGTGGTGAGCCA	TTCAAAATAAGATGGTTCCCCTTA	VIC
LYS	CAACGCTTCTAATTCGATTAGAGGT	GAGCGAAACCCGCATATCC	ACCATCGGCGATAAA	FAM
KAN	CGATGAATCCAGAAAAGCGG	GCTCCTGCCGAGAAAGTATCC	CGCCATGGGTCACG	FAM

Table S15. Bisulfite sequencing, PCR primers.

Target	Primer 1	Primer 2
nosP_bisSeq	GATTATTTGGATTGAGAGTGAATATGAG	TACCCRCCAATATATCCTRTCAAACACT
ChrC_bisSeq	AGAATAAATTAGAAAAGGTGGGGGGGGGG	CCTCCTTTRRATTTATRATTCACCTCAATC

Table S16. Plant lines

Parental	SALK_ID	NASC_ID	Chrom	Start	End	F1
SG260	SALK_126675	N626675	Chr1	22642823.00	22642938.00	SG261/SG368/SG371
SG292	SALK_140062.52.80.x	N640062	Chr1	7458618.00	7458855.00	SG335/SG337SG369
SG298	SALK_112176.43.75.x	N612176	Chr1	22625361.00	22625833.00	SG350/SG361/SG362
SG307	SALK_131115.49.60.x	N631115	Chr1	2952334.00	2952784.00	SG342/SG355/SG356
SG310	SALK_061571.55.25.x	N561571	Chr1	12964622.00	12965035.00	SG340/SG358
SG314	SALK_056646.52.10.x	N556646	Chr1	2085299.00	2085689.00	SG346/SG354/SG357
SG330	SALK_030202.56.00.x	N530202	Chr1	26951796.00	26951870.00	SG352/SG353/SG359
SG333	SALK_058485.56.00.x	N558485	Chr1	17053813.00	17054246.00	SG366/SG367/SG373
SG339	NA	N60000	WT	WT	WT	SG339

Table S17. Aligned read numbers and culture identifiers for all individual 4C experiments.

Viewpoint	aligned reads	4C_replicate	seedling population ID (F1)
KEE6	23189023	KEE6_314	SG354
KEE6	21223451	KEE6_330	SG352
SG260	9685774	SG261_Rep1	SG261
SG260	1378433	SG261_Rep3	SG368
SG260	6521728	SG261_Rep5	SG371
SG260	5405450	SG261_WT1	SG339A
SG260	5784999	SG261_WT2	SG339B
SG260	5735150	SG261_WT3	SG339C
SG292	9913713	SG292_Rep2	SG335
SG292	11091572	SG292_Rep3	SG337
SG292	5100516	SG292_Rep4	SG369
SG292	18036257	SG292_WT1	SG339A
SG292	17736274	SG292_WT2	SG339B
SG292	21479020	SG292_WT3	SG339C
SG298	29828444	SG298_Rep1	SG350
SG298	17469105	SG298_Rep2	SG361
SG298	16456891	SG298_Rep3	SG362
SG298	12474547	SG298_WT1	SG339A
SG298	3740262	SG298_WT2	SG339B
SG298	4485737	SG298_WT3	SG339C
SG307	19553437	SG307_Rep4	SG342
SG307	14139550	SG307_Rep5	SG355
SG307	17357628	SG307_Rep6	SG356
SG307	15779267	SG307_WT4	SG339A
SG307	20777186	SG307_WT5	SG339B
SG307	8076430	SG307_WT6	SG339C
SG310	5530946	SG310_Rep1	SG340*
SG310	5932068	SG310_Rep3	SG358
SG310	10531730	SG310_Rep6	SG340*
SG310	1556512	SG310_WT1	SG339A
SG310	3414811	SG310_WT2	SG339B
SG310	7693095	SG310_WT3	SG339C
SG314	16360973	SG314_Rep1	SG346
SG314	24693231	SG314_Rep2	SG354
SG314	15137004	SG314_Rep3	SG357
SG314	19053104	SG314_WT1	SG339A
SG314	7230100	SG314_WT2	SG339B
SG314	20987454	SG314_WT3	SG339C
SG330	19703711	SG330_Rep2	SG352
SG330	22296675	SG330_Rep3	SG353
SG330	23976923	SG330_Rep4	SG359
SG330	6295082	SG330_WT1	SG339A
SG330	7935708	SG330_WT2	SG339B
SG330	26324223	SG330_WT3	SG339C
SG333	22901701	SG333_Rep2	SG366
SG333	16657521	SG333_Rep3	SG367
SG333	24261975	SG333_Rep5	SG373
SG333	8652969	SG333_WT1	SG339A
SG333	16823014	SG333_WT2	SG339B
SG333	26660207	SG333_WT3	SG339C

Table S18. mRNA sequencing alignment scores

Seedling population ID (F1)	Parental	Aligned reads
SG335	SG292	44403631
SG337	SG292	30517913
SG339B	SG339	26522720
SG339C	SG339	29785015
SG339A	SG339	31035192
SG340A	SG310	30258520
SG340B	SG310	32174764
SG342	SG307	32013397
SG346	SG314	26445754
SG350	SG298	34331765
SG352	SG330	35278271
SG353	SG330	36861323
SG354	SG314	32518373
SG355	SG307	35669341
SG356	SG307	22944868
SG357	SG314	23740897
SG358	SG310	23620741
SG359	SG330	23915939
SG362	SG298	33649195
SG366	SG333	27055650
SG367	SG333	35093779
SG369	SG292	31740768
SG373	SG333	35911536

Table S19. sRNA sequencing alignment scores

Seedling population ID (F1)	reads before filtering	reads after filtering	aligned reads
SG261	7396436	1405063	1311965
SG335	8177634	1344447	1238617
SG337	8334956	1733957	1613459
SG339A	5436747	1070151	1000903
SG339B	8664199	1577092	1469234
SG339C	4745576	1714664	1642939
SG340	8472017	2001790	1876065
SG342	8945031	1698366	1575524
SG346	9513091	1945791	1816128
SG350	9450975	1717736	1592063
SG352	9623763	2063716	1944699
SG353	5413341	1720001	1638880
SG354	7867937	1583755	1475920
SG355	7854309	1731756	1620929
SG356	9235330	1894159	1765857
SG357	7062132	1779930	1680265
SG358	8040484	1527851	1428155
SG359	6522046	994509	923212
SG361	8571275	1559719	1453043
SG362	6945679	1321023	1226693
SG368	7695337	1605855	1503031
SG369	10390466	2234459	2014860
SG371	6860356	1399405	1308957

Supplementary Figures

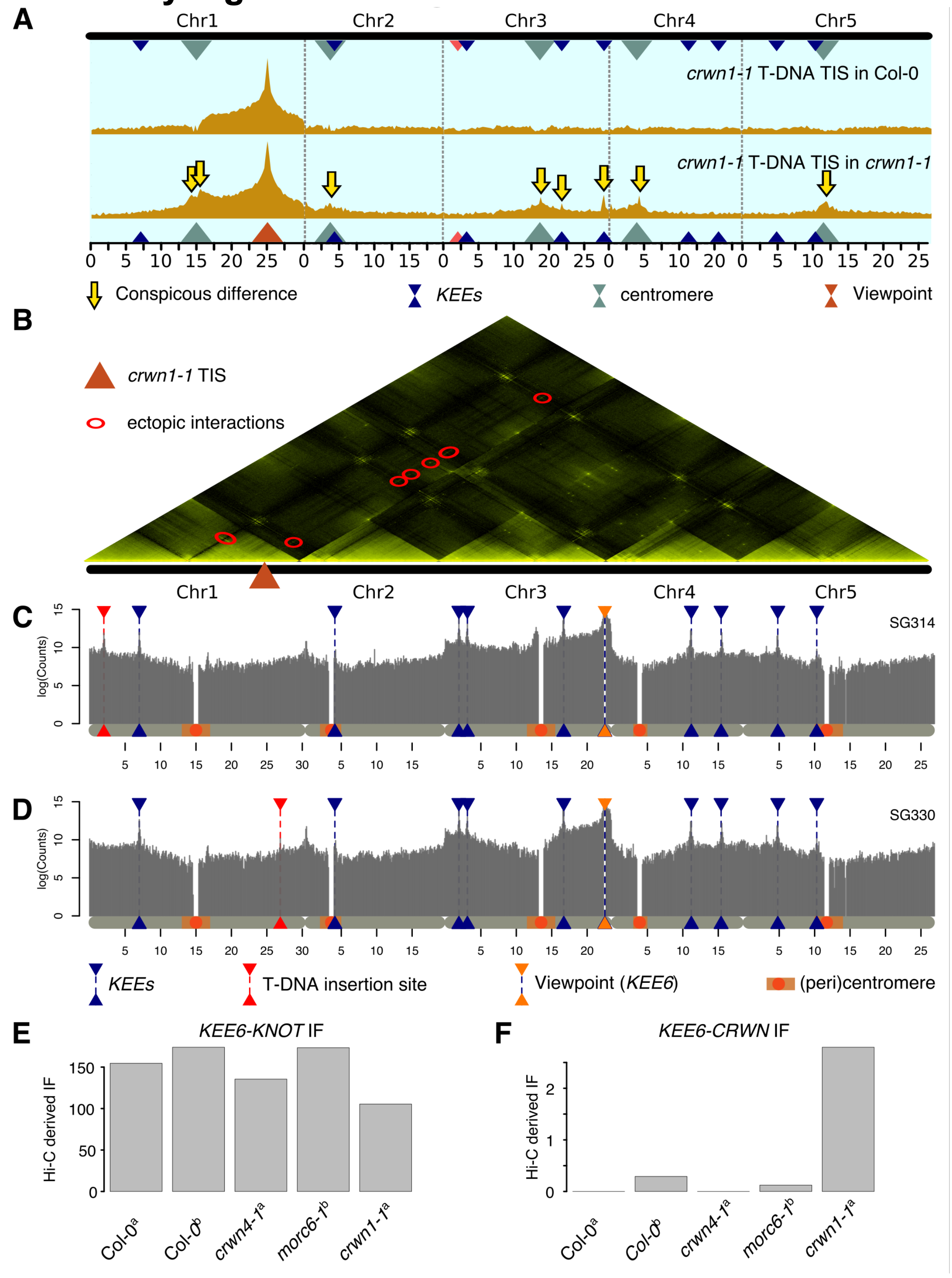

Supplemental Figure S1*.* A) Virtual 4C profile setting the *crwn1-1* TIS as a viewpoint. Top: *in silico* 4C profile extracted from the Col-0 wild-type Hi-C data set. Bottom: *in silico* 4C profile extracted from *crwn1-1* Hi-C data set (Grob et al., 2014). Bin size: 50 kb. Conspicuous differences between transgenic and wild-type virtual 4C profiles are marked with yellow arrows. **B)** Ectopic *KEEs* in *crwn1-1* shown in a Hi-C triangular plot (bin size: 100kb). **C)** 4C profile of viewpoint *KEE6* in SG314. **D)** 4C profile of viewpoint *KEE6* in SG330 **E)** Hi-C derived IFs between *KEE6* and all other *KEEs* in various genotypes. **F)** Hi-C derived IFs between *KEE6* and the *CRWN1* locus. Genotypes in **E)** and **F** (Grob et al., 2014; Moissiard et al., 2012), bin size: 50 kb.

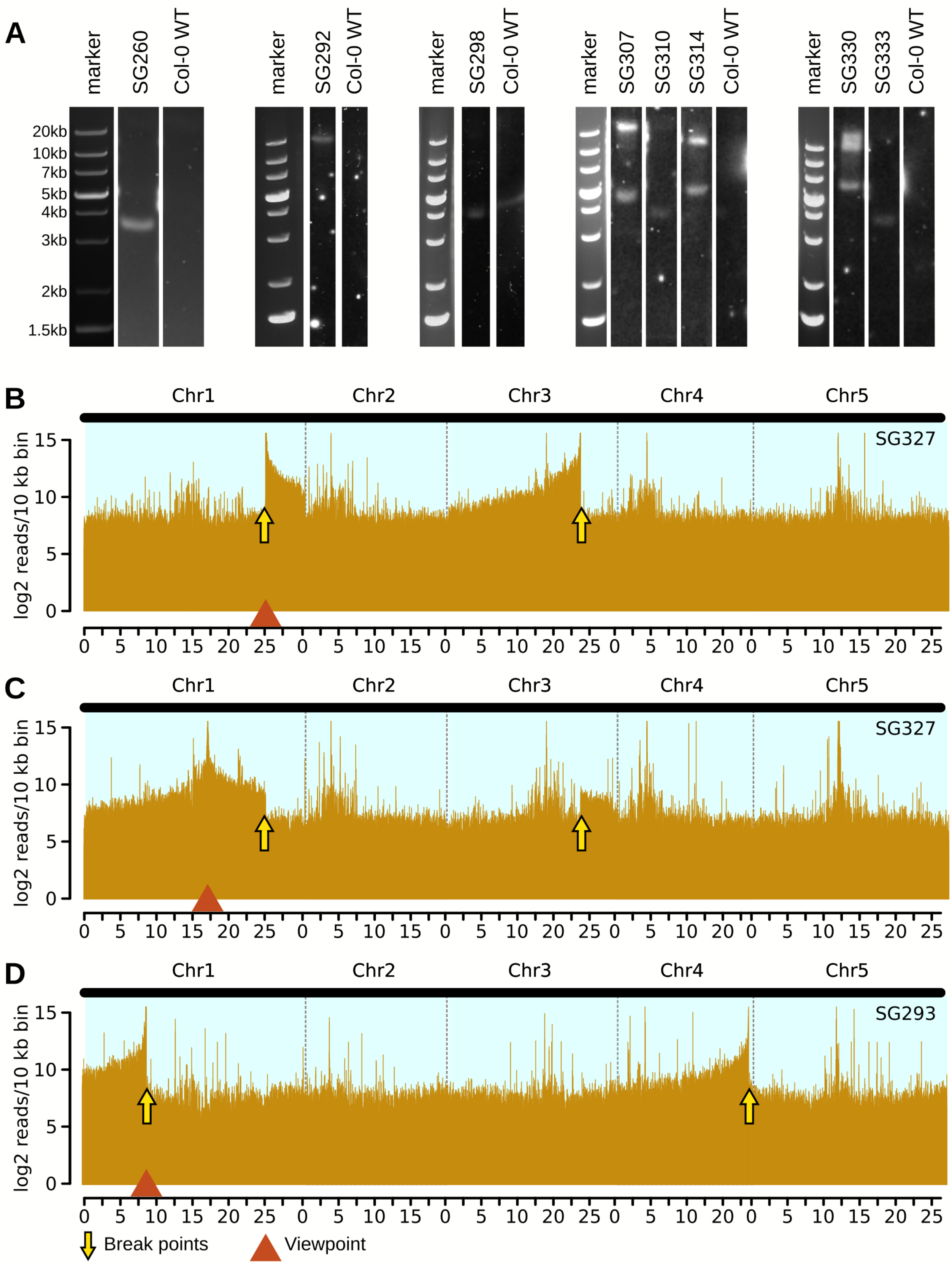

Supplemental Figure S2. A) Southern blot results. Single lanes have been cropped from images containing multiple lanes. DNA of plant samples non-relevant for the present study where loaded on these lanes. **B)-C)** Detection of translocations by 4C. **B)** 4C interaction frequencies from a viewpoint in close proximity of the TIS of SG327. A translocation occurred between chromosome 1 and chromosome 3. **C)** 4C interaction profile obtained from the same transgenic line as in B) with a different viewpoint. **D)** 4C interaction frequencies from a viewpoint in proximity of the TIS of SG293. A translocation occurred between chromosome 1 and chromosome 4.

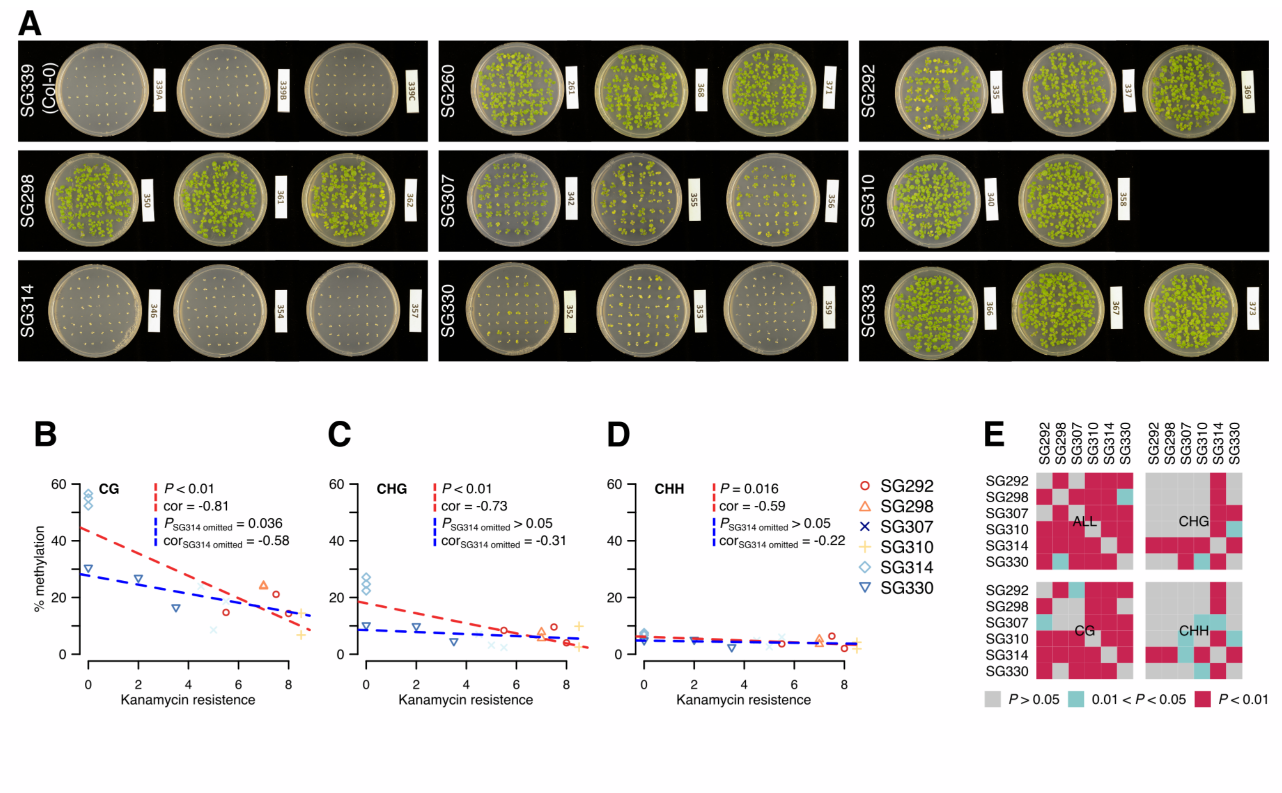

Supplemental Figure S3*.* A) Kanamycin resistance assay. 14-day-old seedlings grown on medium containing kanamycin. Parental line identified is indicated on the left. **B) - E)** Pearson's correlation analysis between kanamycin resistance phenotype and *nosP* methylation levels. **B)** CG context, **C)** CHG context, **D)** CHH context. Red dashed lines show correlation including all six transgenic lines, blue dashed lines omit SG314 in analysis. **E)** Summary of significance of cross-wise Chi-square testing between transgenic lines, split for individual cytosine methylation contexts.

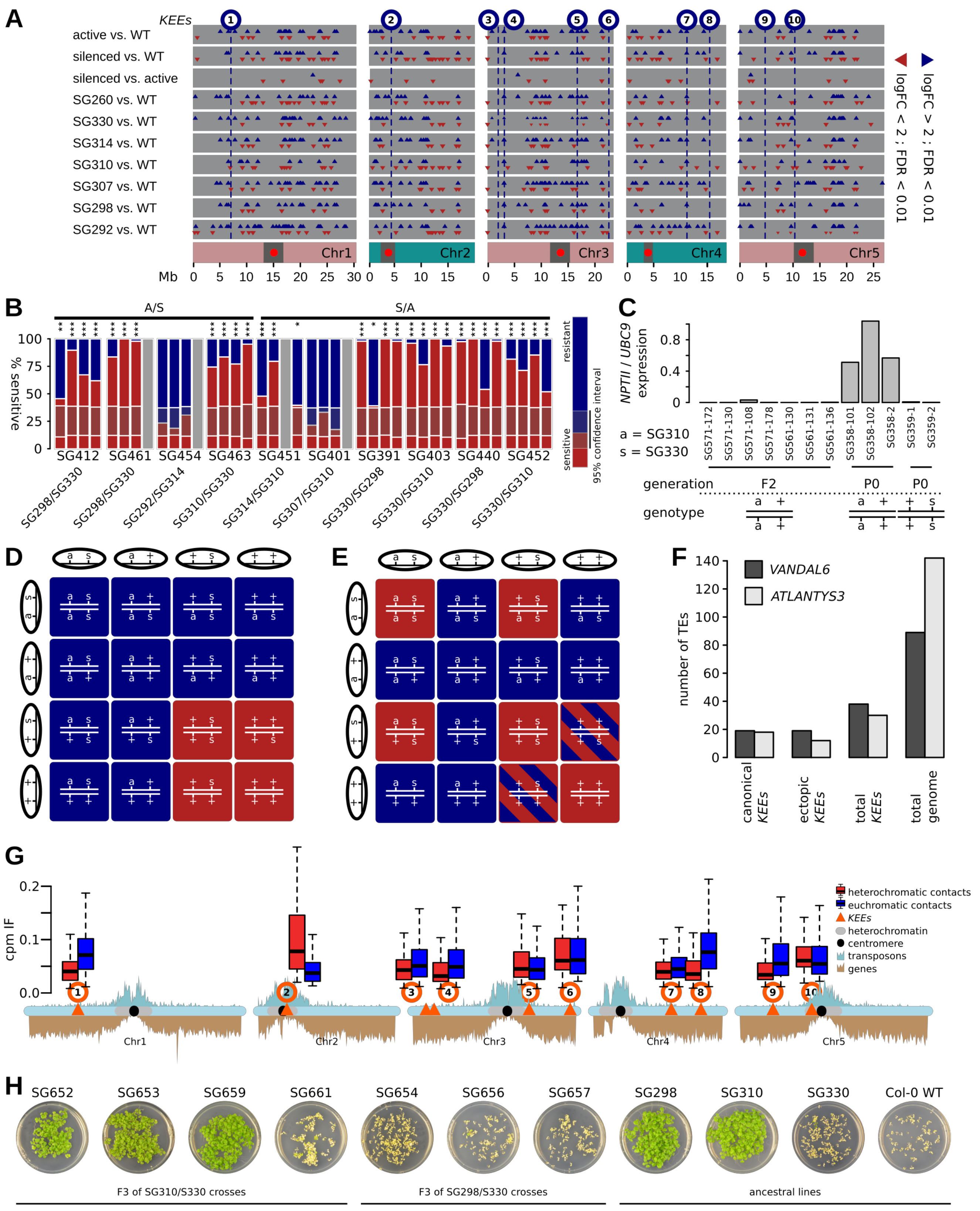

A
KEEs
active vs. WT
silenced vs. WT
silenced vs. active
SG260 vs. WT
SG330 vs. WT
SG314 vs. WT
SG310 vs. WT
SG307 vs. WT
SG298 vs. WT
SG292 vs. WT
Chr1 Chr2 Chr3 Chr4 Chr5
Mb
logFC > 2 ; FDR < 0.01
logFC < 2 ; FDR < 0.01
B
A/S
S/A
% sensitive
SG412 SG461 SG454 SG463 SG451 SG401 SG391 SG403 SG440 SG452
SG298/SG330 SG298/SG330 SG292/SG314 SG310/SG330 SG314/SG310 SG307/SG310 SG330/SG298 SG330/SG310 SG330/SG298 SG330/SG310
resistant
sensitive
95% confidence interval
C
NPTII / UBC9 expression
a = SG310
s = SG330
SG571-172 SG571-130 SG571-108 SG571-178 SG561-130 SG561-131 SG561-136 SG358-101 SG358-102 SG358-2 SG359-1 SG359-2
generation F2 P0 P0
genotype
D
E
F
VANDAL6
ATLANTYS3
number of TEs
canonical KEEs
ectopic KEEs
total KEEs
total genome
G
cpm IF
heterochromatic contacts
euchromatic contacts
KEEs
heterochromatin
centromere
transposons
genes
Chr1 Chr2 Chr3 Chr4 Chr5
H
SG652 SG653 SG659 SG661 SG654 SG656 SG657 SG298 SG310 SG330 Col-0 WT
F3 of SG310/S330 crosses
F3 of SG298/S330 crosses
ancestral lines

Supplemental Figure S4. A) sRNA-seq overview on various contrasts for which differential analysis was performed. Genomic bins (1 kb) associated with significant changes (logFC > 2; FDR < 0.01) are shown as triangles (blue, upregulation; red, downregulation). **B)** Representation of the segregation analysis of F_2 seedling populations from individual F_1 mothers. Chi-square tests were performed to test for deviation from Mendelian segregation (Null-hypothesis: 0.25/0.75 (sensitive/resistant), *: 0.05 > P ≥ 0.01, **: 0.01 > P ≥ 0.001, ***: P < 0.001). Confidence interval indicates the range, in which Mendelian segregation cannot be rejected. A: active ancestral phenotype, S: silenced ancestral phenotype. **C)** Kanamycin expression in F_2 and parental P0 plants assessed by ddPCR. **D)** Expected genotype-phenotype relationship assuming Mendelian segregation of phenotypes in the F_2 generation. **E)** Expected genotype-phenotype relationship assuming a dosage effect of an interfering small RNA produced by the S transgene. sRNA dosage may explain, why in the F_1 generation (silenced allele in hemizygous state) no *trans*-silencing effect can be observed but only in the F_2 generation. However, ratios higher than the expected ratio of kanamycin sensitive to kanamycin resistant (5/16 – 7/16) can be observed in the F_2 generation (see **C)**) a: active allele, s: silenced allele, circles: maternal (top) and paternal (left) gametes, red: kanamycin sensitive phenotype, blue: kanamycin resistant phenotype. **F)** Abundance of *VANDAL6* and *ATLANTYS3* TEs in canonical and ectopic (induced by *ddm1* - (Feng et al., 2014)) *KEEs*. **G)** Boxplot of the distribution of contact frequencies separated for hetero- (red) and euchromatic (blue) partners for individual *KEEs* (only considering *trans*-contacts; data extracted from 50 kb Hi-C bins (Grob et al., 2014)). **H)** 14-day-old F_3 seedlings grown on kanamycin-containing culture medium. The F_3 seedling populations derive from single silenced F_2 plants, which were homozygous for the A-transgene and lacked the S-transgene.

References

Feng, S., Cokus, S.J., Schubert, V., Zhai, J., Pellegrini, M., and Jacobsen, S.E. (2014). Genome-wide Hi-C analyses in wild-type and mutants reveal high-resolution chromatin interactions in *Arabidopsis*. Mol. Cell *55*, 694–707.

Grob, S., Schmid, M.W., and Grossniklaus, U. (2014). Hi-C Analysis in *Arabidopsis* identifies the *KNOT*, a structure with similarities to the flamenco locus of *Drosophila*. Mol. Cell *55*, 678–693.

Moissiard, G., Cokus, S.J., Cary, J., Feng, S., Billi, A.C., Stroud, H., Husmann, D., Zhan, Y., Lajoie, B.R., McCord, R.P., et al. (2012). MORC family ATPases required for heterochromatin condensation and gene silencing. Science. *336*, 1448–1451.
